# Impact of Stereocontrolled Polynorbornene Synthesis on Degradation Rate

**DOI:** 10.1021/acs.macromol.5c01049

**Published:** 2025-08-29

**Authors:** Britney Baez, Greyson Karis-Sconyers, Ethan Flanagan, Aech Loar, Spencer D. Brucks

**Affiliations:** Department of Chemistry, 31750 Harvey Mudd College, Claremont, California 91711, United States

## Abstract

We synthesized a family of polynorbornenes with a wide range of *cis*/*trans* compositions by ring-opening metathesis polymerization (ROMP) with a variety of ruthenium catalysts and subjected these materials to ultrasonication as a proxy for mechanical degradation. Increasing the *cis*-alkene content enhanced the degradation rate for both short (∼70 kDa) and long (∼700 kDa) polynorbornenes. These data suggest that the heightened allylic strain in *cis*- versus *trans*-polynorbornene promotes faster degradation independent of molecular weight under mechanical stress. Our results complement existing plastics recycling research by isolating the understudied relationship between backbone stereochemistry and degradability.

## Graphical abstract

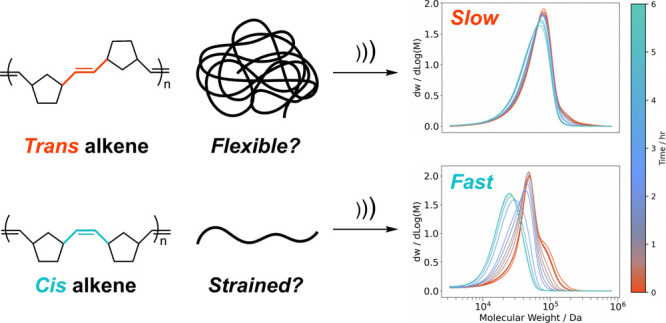

## Introduction

Even as annual global plastic production exceeds 400 million metric tons, plastic recycling has remained stubbornly below 10% of all plastic waste.^1^ Viable strategies for this plastic crisis are desperately needed. Significant research efforts have focused on the development of novel bioplastics derived from renewable feedstocks such as starch, lignin, or cellulose.^2,3^ These efforts are commendable but have yet to make a commercial impact, with the most prominent bioplastic—polylactide—accounting for only 0.1% of global plastic production.^4^ Other approaches have focused on varying the polymer composition to imbue degradability by design.^5^ For example, the incorporation of silyl ether monomers was shown to imbue acid degradability,^6^ while installation of simple ethers^7^ and ketones^8^ imparted photodegradability. Similar compositional modifications have enhanced polymer degradation by pyrolysis^9^ and enabled catalytic^10^ or enzymatic^11^ depolymerization. While these methods offer important, creative solutions, they do not readily translate to current industrial infrastructure that primarily leverages mechanical degradation for plastics reprocessing.^12^ Thus, there is a tremendous demand for understanding the mechanical degradability and consequential chain scission of commercially relevant plastics.

The mechanical degradation of polymers has been studied in dendritic,^13^ star,^14^ cyclic,^15^ and bottlebrush^16^ systems, and these studies collectively suggest that degradation rate increases with polymer chain elongation.^17^ In bottlebrush systems, degradation kinetics have been shown to be a function of polymer stiffness, where increasing either grafting density or arm length promotes a more extended conformation that undergoes faster degradation by ultrasonication.^18^ Other studies have indicated that solvent choice alone can impact polymer conformations in solution and that solvents that promote an extended conformation similarly undergo more rapid degradation by ultrasonication.^19,20^ This ultrasonication technique is commonly used as a proxy for mechanical degradation because cavitation generates shearing forces that can lead to the midchain cleavage of polymers of sufficient length (>30 kDa).^21,22^ While the ultrasonic degradation of polymers has been employed for nearly a century,^23,24^ and many structure–function investigations have been conducted,^17^ the impact of the polymer backbone has been largely underexplored.

A recent study suggested that the polymer backbone may play an important role in dictating overall chain conformation and function.^25^ Polynorbornenes functionalized with galactose were found to adopt an extended rod-like conformation when the backbone was enriched in *cis*-alkenes compared to a globular spherelike structure when primarily composed of *trans*-alkenes. This morphological effect was rationalized by the increased allylic strain in a *cis*-polynorbornene dimer relative to that in the *trans* configuration. While previous studies have shown that *cis*-polynorbornene has a marginally higher glass transition and thermal decomposition temperature than *trans*-polynorbornene,^26,27^ this was one of the first studies demonstrating a functional difference between the two stereoisomers. Given these potential morphological differences between *cis-* and *trans*-polynorbornene and recent works suggesting that *cis*-stereoisomers transduce force more effectively than their *trans* analogues,^28,29^ we were curious if backbone alkene stereoisomerism would have any consequences on mechanical degradation.

We hypothesized that increasing allylic strain in a polyalkenamer by enriching the *cis*-alkene content in the backbone would enhance the polymer’s susceptibility to mechanical shearing forces. For this study, we chose to focus on polynorbornene, which has a cyclopentane embedded in its backbone that results in a significant allylic strain difference between the *cis* and *trans* configurations. Furthermore, norbornene is a prototypical monomer for ring-opening metathesis polymerization (ROMP), yielding an aliphatic polymer without functional groups that could conceivably impact polymer stiffness.^30^ We therefore synthesized polynorbornenes with a range of stereochemical backbone compositions by ROMP using a variety of Grubbs-type catalysts (Figure 1) and then studied their rate of mechanical degradation by ultrasonication. The resulting degradation profiles matched our prediction that increasing the *cis*-alkene content in polynorbornene increases the degradation rate, even for polymers with similar initial molecular weights. Our results suggest that the polymer backbone can serve as a surprising handle for tuning the degradability of polynorbornenes.

**Figure 1:**
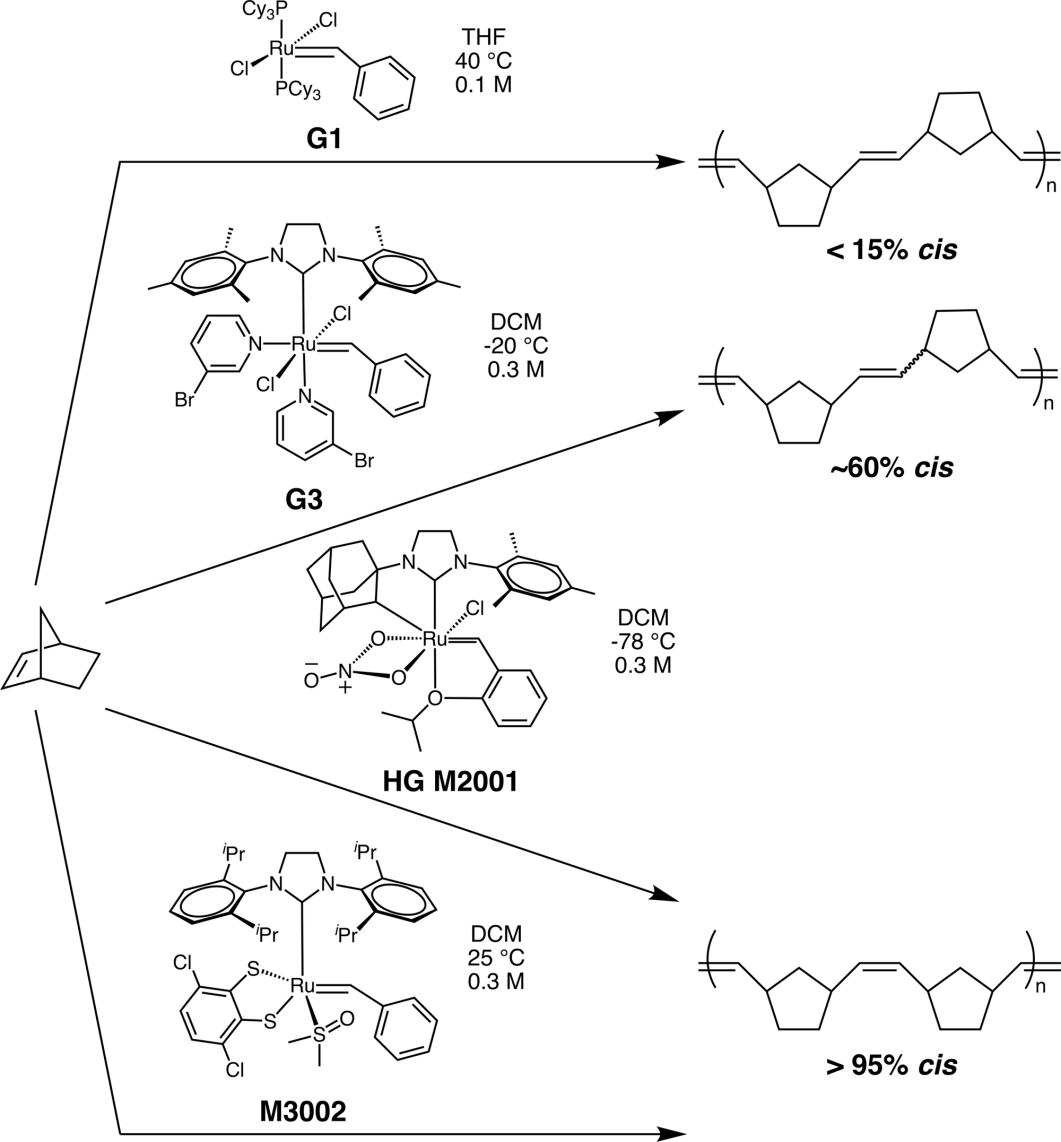
Synthetic scheme for polynorbornenes with varied *cis*-alkene content.

## Results and Discussion

We synthesized a library of polynorbornenes at two different molecular weights that varied only in the stereochemistry of their olefinic backbone (Table 1). We accessed a wide range of *cis*-alkene content (∼10%–100%) by varying the Grubbs-type ruthenium catalyst involved in the ROMP reaction. The *cis*-alkene content of these polymers was quantified by comparing the relative integrations of the ^1^H NMR peaks corresponding to the allylic protons at δ 2.79 ppm (*cis*-alkene) and δ 2.43 ppm (*trans-*alkene) as previously described (Figures S1–S7).^31,32^ After optimization of the reaction conditions for each catalyst (Table S1), we found that ROMP with the first generation Grubbs catalyst (G1) yielded highly *trans*-enriched polymers (∼10% *cis*-alkene content, Table 1, entries 1 and 2), while ROMP with the third generation Grubbs (G3) yielded slightly *cis*-enriched polymers (∼60% *cis*-alkene content, Table 1, entries 3 and 4). Τwo different Grubbs-type catalysts (Hoveyda–Grubbs (HG) M2001 and Grubbs M3002) were employed to afford highly *cis*-enriched polymers (∼99% *cis*-alkene content, Table 1, entries 5 and 6).

**Table 1:** Library of Synthesized Polynorbornenes with Varied *cis*-Alkene Content

entry	catalyst	M/I/Cl_2_Py	temp (°C)	conc (M)	time (min)	yield (%)	*M*_*n*_ (kDa)^a^	*M*_*w*_ (kDa)^a^	*Đ* ^a^	*Cis*-alkenes (%)^b^
**1a**	G1	5000/1/0	40	0.1	10	94	560	740	1.33	11
**1b**	G1	5000/1/0	40	0.1	15	92	640	900	1.40	12
**1c**	G1	5000/1/0	40	0.1	10	92	550	890	1.60	11
**2a**	G1	500/1/0	40	0.1	10	92	60	85	1.36	11
**2b**	G1	500/1/0	40	0.1	10	89	55	75	1.36	12
**2c**	G1	500/1/0	40	0.1	10	86	50	75	1.49	12
**3a**	G3	5000/1/0	–20	0.3	30	92	360	510	1.41	61
**3b**	G3	8000/1/0	–20	0.3	30	91	420	580	1.37	62
**3c**	G3	10000/1/0	–20	0.3	30	96	520	710	1.38	62
**4a**	G3	500/1/0	–20	0.3	15	92	50	60	1.09	61
**4b**	G3	500/1/0	–20	0.3	15	97	65	75	1.16	61
**4c**	G3	500/1/0	0	0.3	15	82	60	70	1.22	61
**5a**	M2001	500/1/0	–78	0.3	30	53	430	680	1.58	98
**5b**	M2001	500/1/0	–78	0.3	20	51	660	1070	1.63	98
**5c**	M2001	500/1/0	–78	0.3	20	44	400	720	1.76	98
**6a**	M3002	500/1/12.5	20	0.3	10	56	45	55	1.16	99
**6b**	M3002	500/1/12.5	20	0.3	10	84	45	60	1.34	100
**6c**	M3002	500/1/12.5	20	0.3	10	86	60	90	1.51	100

a Determined by size exclusion chromatography in THF using polystyrene standards and RI detection.

b Determined by ^1^H NMR (400 MHz, CDCl_3_).

Because longer polymer chains are known to degrade faster than shorter chains by ultrasonication,^33^ we suspected a potential interplay between polymer size and backbone stereochemistry in the degradation kinetics of our polymers. We therefore targeted two distinct degrees of polymerization (DP), 5000 and 500, for each of the three stereochemical compositions in order to test the effect of backbone stereochemistry on degradation rate independent of DP. Additionally, we synthesized three independent samples for every unique polymer profile with a given *cis*-alkene content and DP (Table 1, a–c for each entry) to verify the reproducibility of our synthetic methods and ultimately perform degradation experiments in triplicate of an approximate *cis*-alkene % and *M*_w_.

As has been previously reported, ROMP with HG M2001 exhibits poor molecular weight control,^34,35^ and we were unable to synthesize polynorbornenes with fewer than 1000 repeat units using this catalyst. Indeed, when targeting a DP of 500, we consistently obtained polymers with DP ∼5000. We therefore sought to find an alternative catalyst with equally high *cis*-selectivity and were intrigued by recent reports of a sulfinyl-containing, stereoretentive Grubbs catalyst (M3002) that polymerized norbornene derivatives with remarkable *cis*-selectivity and molecular weight control.^35,36^ Gratifyingly, norbornene polymerization with this catalyst in the presence of 2.5 mol % 3,5-dichloropyridine (Cl_2_Py) afforded highly *cis*-enriched polymers (up to 99% *cis*) of the desired molecular weight with satisfactory dispersities (Table 1, entry 5). We note that this reaction proceeded poorly in the absence of Cl_2_Py, which is reported to slow polymer propagation by stabilizing the carbene intermediate,^37^ and that M3002 is highly oxygen sensitive and must therefore be handled in a glovebox.

In preparing our polynorbornene library, we found that increasing the ROMP reaction temperature increases the dispersity while decreasing the *cis*-alkene content of polymers synthesized with the third generation Grubbs (G3) catalyst in dichloromethane (Figure 2). While the G3 catalyst is widely used for ROMP, the effects of the reaction temperature on dispersity and *cis*-alkene content are poorly consolidated in the literature. Here, we observed a drastic dependence of the dispersity on reaction temperature, with *Đ* as low as ∼1.20 at −78 and −20 °C, but then increasing dramatically to *Đ* ∼3.0 at 35 °C. Reaction temperature effects on the *cis*-alkene content were more subtle, with G3-catalyzed reactions consistently yielding 61% *cis*-alkenes at temperatures below 0 °C but decreasing to 58% at elevated temperatures. The observed trends were largely generalizable to polymerizations conducted with HG M2001, which also required colder temperatures to achieve the lowest dispersities but yielded high *cis*-alkene content at all tested temperatures (Table S1, entry 2). We were surprised to find that polymerizations with G1 required heating to achieve the lowest dispersities, which were accessed at an optimal temperature of 40 °C (Table S1, entry 3).

**Figure 2:**
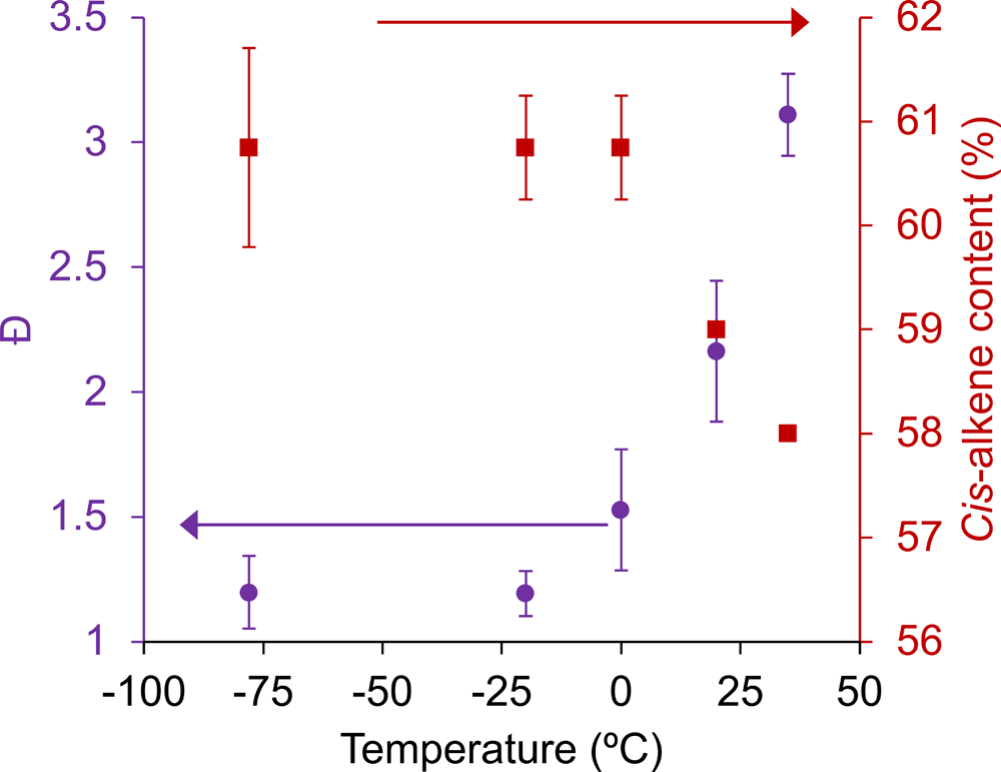
ROMP reaction temperature effects on polymer dispersity (*Đ*) and *cis*-alkene content for the polymerization of norbornene with G3 in DCM at 0.3 M. Data are presented as the average of triplicate measurements from three distinct samples, with error bars representing the standard deviation of the mean.

With our library of polynorbornenes of varying lengths and *cis*-alkene content in hand, we next sought to investigate the relationship between polymer backbone stereochemistry and degradability. Triplicate samples of each unique polynorbornene profile were dissolved in tetrahydrofuran (THF) and subjected to 6 h of continuous ultrasonication at 37 kHz and ∼0.4 W cm^–2^ and a constant temperature of 26 °C. Aliquots of the degradation products were taken intermittently and characterized by gel permeation chromatography (GPC) to track changes in the molecular weight distribution of the sample over sonication time (Figure 3, Tables S2–S7). We visualized decreases in the average molecular weight by overlaying the GPC traces of the original sample and every degradation aliquot (Figures S8–S19). Directly comparing polymers that were synthesized with similar *M*_w_ ∼700 kDa and dispersity (namely polymers **1c**, **3c**, and **5a**) revealed that the *trans*-enriched and *cis*-enriched samples break down to distinct chain lengths after 6 h of ultrasonication (Figure 3A). While the *trans*-enriched polymer **1c** (∼11% *cis-*alkenes) broke down to *M*_w_ ∼70 kDa (Table S2), the *cis*-enriched polymer **5a** (∼98% *cis*-alkenes) broke down to less than one-third of this value, *M*_w_ ∼20 kDa (Table S6). Polymer **3c** with ∼60% *cis*-alkenes broke down to an intermediate *M*_w_ of ∼30 kDa (Table S4).

**Figure 3:**
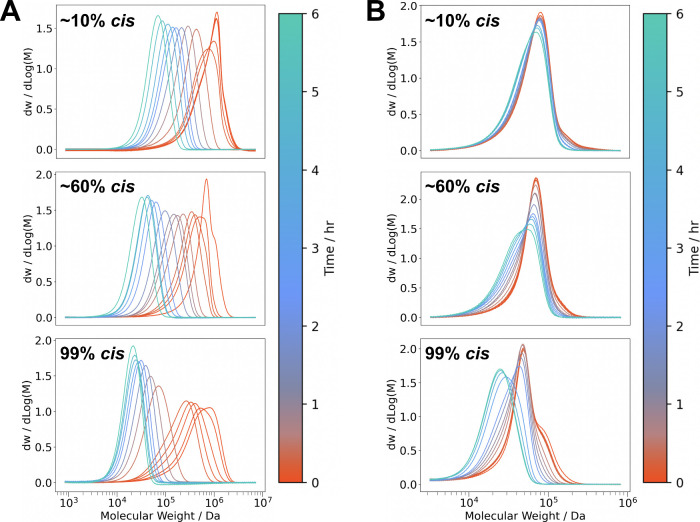
Overlaid molecular weight distributions of long and short polynorbornene samples over 6 cumulative hours of ultrasonic degradation for (A) DP = 5000 (top to bottom: polymers **1c**, **3c**, **5a**) and (B) DP = 500 (top to bottom: polymers **2b**, **4c**, **6b**).

Given that the *cis*-alkene content could impact polymer morphology, it may also affect GPC retention time and apparent molecular weight. Because cyclic polymers are known to have a smaller hydrodynamic volume than their corresponding linear polymers,^38^ a *trans*-enriched sample that adopts a more compact, globular structure may have a longer retention time than a *cis*-enriched sample with a more elongated structure and larger hydrodynamic volume. If this were indeed the case, then our GPC data of the parent polymers may be overestimating the initial molecular weight of a more linear *cis*-enriched polymer and underestimating the initial molecular weight of a more globular *trans*-enriched polymer. Similarly, GPC analysis may be overestimating fragmentation in *trans*-enriched samples and underestimating fragmentation in *cis*-enriched samples, suggestive of an even greater difference in the degradation rate than reported herein. However, we suspect these GPC analysis effects are minimal because the reported molecular weights are consistent with theoretical molecular weights based on the monomer to catalyst loading and known molecular weight control of each catalyst.^34^

Because ultrasonic shearing forces promote midchain cleavage yielding polymer fragments with half their original DP (1/2 daughters), successive chain scission events should yield fragments of approximately 1/4 and 1/8 the original DP (1/4 daughters, and 1/8 daughters, respectively).^13,22^ We therefore analyzed the degradation of our samples by how quickly the peak molecular weight (*M*_p_) shifted to these daughter fragments (Figure S20). While the *trans*-enriched polymer **1c** required >25 min of ultrasonication for its *M*_p_ to reach the 1/2 daughter fragment, the *cis*-enriched polymer **5a** achieved this fragmentation in only 3 min. This contrast was amplified for the downstream fragments, with the *trans*-enriched sample requiring ∼210 min of ultrasonication to reach the 1/8 daughter, while the *cis*-enriched sample reached it in less than 25 min—again, an order of magnitude faster.

We further performed scission cycle (SC) calculations, using the method developed by Craig and co-workers,^19^ to measure the generation of daughter fragments, which further underscored the distinction between *trans*- and *cis*-enriched polynorbornenes (Figures S21–S28). While the *trans*-enriched sample only reached its third scission cycle (SC = 3.38), indicative of its 1/8 daughter, after 6 h of ultrasonication, the 60% *cis* sample reached its fourth scission cycle (SC = 4.40), indicative of its 1/16 daughter. The *cis*-enriched sample also exceeded its fourth scission cycle and nearly reached its fifth scission cycle (SC = 4.65) after the full 6 h.

Next, we wanted to test how these results would translate to shorter polynorbornenes with only 500 repeat units (*M*_w_ ∼70 kDa). We repeated our procedure of dissolving our polymers in THF and subjecting them to 6 h of continuous ultrasonication with aliquots taken intermittently. Gratifyingly, comparing the overlaid molecular weight distribution curves of polymers with similar initial *M*_w_, namely, **2b**, **4c**, and **6b**, corroborated that increased *cis*-alkene content in the polymer backbone leads to enhanced degradation and a smaller final *M*_w_ (Figure 3B). Given that ultrasonic degradation is significantly slower for polymer chains <100 kDa,^21^ fewer scission cycles and daughter fragments were produced than for the 5000-mers. Nevertheless, we were surprised by the stark contrast between the *trans*-enriched and *cis*-enriched degradation profiles and scission cycle analyses (Figures S22, S29–S34). After 6 h of ultrasonication, the *trans*-enriched polymer **2b** barely broke down from *M*_w_ ∼75 kDa to *M*_w_ ∼ 60 kDa (SC = 0.32), while the *cis*-enriched polymer **6b** degraded to a *M*_w_ of ∼25 kDa (SC = 1.12). The high-molecular weight shoulder was the first to be degraded in both samples, which is consistent with ultrasonic degradation targeting longer chains first. While this is the only degradation the *trans*-enriched polymer exhibited after 6 h, the *cis*-enriched polymer lost its high molecular weight shoulder within 45 min, and then the entire molecular weight distribution shifted toward the 1/2 daughter. We only observed a similar fragmentation to the 1/2 daughter in the *trans*-enriched polymer after 24 h of continuous ultrasonication (Table S8, entry 1). Even then, after the same 24 h sonication treatment, the *cis*-enriched polymer reached a *M*_w_ of ∼15 kDa (Table S8, entry 3), indicative of its 1/4 daughter fragment. This contrast between the *M*_w_ values of the *cis*- and *trans*-enriched samples after this extended sonication treatment suggests that the molecular weight limit of degradation may be a function of *cis*-alkene content.

To confirm that polymer degradation was driven exclusively by ultrasonic shearing forces and not localized heating or radical generation, we additionally synthesized polymers with a *M*_w_ of ∼25 kDa matching the theoretical molecular weight limit of degradation.^21^ Simply modifying the monomer to catalyst ratio gave us straightforward access to polynorbornenes of the desired *M*_w_ with reasonable *Đ* and *cis*-alkene content ranging from 10–100% (Table S9). We then repeated our dissolution in THF and sonication procedure for 6 continuous hours. We found that the *trans*-rich and intermediate compositions exhibited no degradation after 6 h of sonication, suggesting that they were indeed too small to be degraded by shearing forces and that no additional stimuli were promoting degradation (Figures S35 and S36). However, we were surprised to find that the 100% *cis*-polymer partially degraded with a significant low molecular weight shoulder visible after 6 h of sonication (Figure S37). We hypothesized that this evident degradation may be due to the increased *cis*-alkene content lowering the molecular weight limit of degradation. Therefore, we synthesized one additional *cis*-enriched polymer with a *M*_w_ of ∼12.5 kDa and subjected it to our standard degradation procedure. We were pleased to find that this polymer did not exhibit any degradation, suggesting both that no additional stimuli contributed to the *cis*-polymer degradation and that the molecular weight limit of degradation of these *cis*-enriched polynorbornenes is likely closer to 12.5 kDa (Figure S38). As a final control, we found that our polymers did not exhibit degradation in THF in the absence of ultrasonication (Table S10), further suggesting that the observed degradation is driven exclusively by ultrasonic shearing forces and that susceptibility to degradation may be a consequence of backbone stereochemistry.

We also did not observe significant alkene isomerization by ^1^H NMR as a result of our ultrasonication (Figures S39–S44). Previous studies of polyoxanorbornene instead observed a rapid isomerization during single molecule force spectroscopy.^39,40^ This discrepancy might be due to time scale or thermal effects or the fact that only a small fraction of the polymer midchain is susceptible to isomerization during ultrasonication.^41^ Indeed, it is possible that a small fraction of *cis*-alkenes are isomerizing during ultrasonication, but due to the large number of repeat units, the resulting change is imperceptible by ^1^H NMR.

In order to quantify the polymer degradation rates of the three stereochemical compositions, we analyzed the concentration decrease of the initial peak molecular weight (M_p_) intensity over the sonication time (Figure 4). Because scission rate is known to be a function of molecular weight, this method allows for quantification of the decay of a single molecular weight over time.^42,43^ We first normalized all samples by peak area to account for concentration differences, and then, each sample was normalized by its parent M_p_ intensity to ensure that the signal intensity at t = 0 min was equal to 1.0 (Figures S45–S56). Analysis of the same 5000-mers presented in Figure 3A (**1c**, **3c**, and **5a**) revealed that the intensity of the initial M_p_ decreased the fastest for the *cis*-enriched polymer (Figure 4A). After 10 min of ultrasonication, the residual intensity of the *cis*-enriched polymer’s initial M_p_ was less than 10%, while more than half of the *trans*-enriched polymer’s initial M_p_ intensity remained. The 500-mers exhibited a similar trend, with the *cis*-enriched polymer’s initial M_p_ decaying to the greatest degree after 6 h of ultrasonication (Figure 4B). While all 5000-mer samples reached a normalized M_p_ intensity of zero within ∼30 min of sonication, all of the 500-mers still possess a nonzero intensity after 6 h, highlighting the strong dependence of scission rate on molecular weight. Indeed, the ∼99% *cis-*500-mer exhibits a slower decrease in M_p_ intensity than the 60% *cis-*500-mer in the first ∼150 min of sonication because of the degradation of its high-molecular weight shoulder (Figure S57).

**Figure 4:**
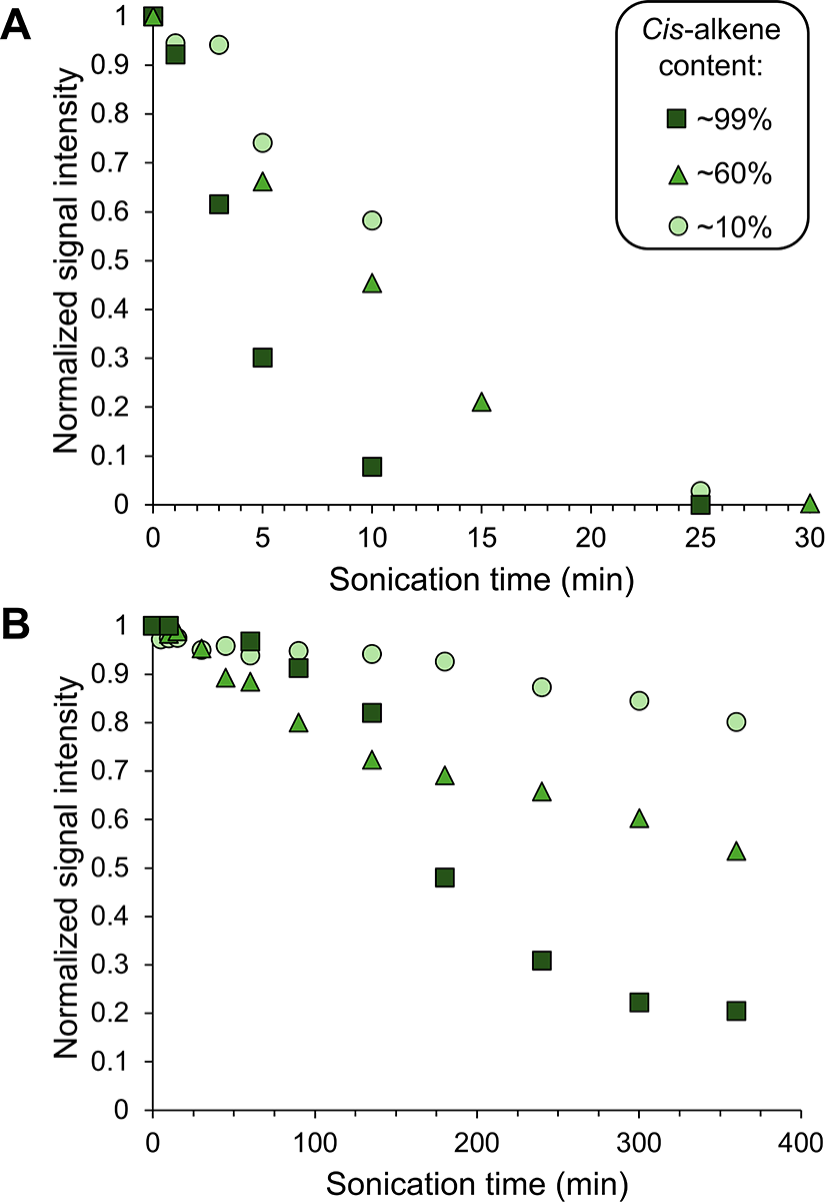
Normalized refractive index signal intensity of the initial peak molecular weight, *M*_p_, in the molecular weight distribution for polynorbornenes of both (A) DP = 5000 (∼10% *cis*-alkenes **1c**, ∼60% *cis*-alkenes **3c**, and ∼99% *cis*-alkenes **5a**) and (B) DP = 500 (∼10% *cis*-alkenes **2b**, ∼60% *cis*-alkenes **4c**, and 99% *cis*-alkenes **6b**) over 6 cumulative hours of ultrasonic degradation.

Boydston and Peterson similarly report that this intensity analysis method is sensitive to GPC conditions and polymer dispersity, with higher-dispersity samples yielding less reliable results.^43^ Ideally, each intensity curve could be fit to exponential decay functions that would enable extraction of an exponential decay rate for the starting DP, but the presence of a high-molecular weight shoulder precludes a clean analysis. We were therefore motivated to explore additional methods for comparing the degradation kinetics of our polymers.

To further compare the degradation kinetics of our polynorbornenes, we sought to calculate decay rates for each stereochemical composition as a function of the degree of polymerization and *cis*-alkene content. While there does not seem to be a universal method for modeling polymer degradation kinetics, several methods have been developed and validated. The Malhotra model assumes a linear relationship between scission rate and molecular weight^44,45^ and has been applied to a variety of materials undergoing midchain scission.^29,46^ However, this model is less suitable for large polymers with daughter fragments that can undergo further decay.^43^ Robb and co-workers recently validated an initial rates method to deconvolute chain scission from mechanophore activation,^47^ but the scission of our polynorbornenes is not in competition with mechanophore activation or another pathway. Instead, we are focused on isolating the effect of backbone composition on degradation rate as opposed to the dependence of scission rate on polymer DP. We therefore opted for an exponential decay model of the form MW = *A*(1 – *r*)^*t*^ + *c*, which allowed us to extract a single exponential decay rate, *r*, for each polymer composition. While this model assumes a constant decay rate over the period of ultrasonication, it enables pairwise comparisons between the three stereochemical compositions in polynorbornenes with similar initial degrees of polymerization and dispersity, thus isolating the effect of backbone stereochemistry on the degradation rate.

We fit the *M*_n_ and *M*_w_ values of each polymer sample and their degradation products over the 6 h of sonication to exponential decay curves and extracted a decay rate for each (Figures S58–S75). All degradations were well-fit by the model (*R* ^2^ > 0.89; most *R* ^2^ > 0.97). We then calculated the average decay rate (Figure 5) for each unique polymer profile from both the *M*_n_ and the *M*_w_ fittings. Focusing on the *M*_n_ analysis, we found that highly *cis*-enriched polynorbornenes have the greatest decay rate among both the 5000- and 500-mers. All three stereochemical compositions of the 5000-mers have a high average decay rate (*r* > 0.88 min^–1^), which increases with *cis*-alkene content and reaches a remarkable and consistent decay rate of ∼0.99 min^–1^ in the samples with a fully *cis* backbone independent of exact *M*_n_ or dispersity. This rapid decay rate of the *cis*-enriched polynorbornenes is significantly different from that of the *trans*-enriched 5000-mers (*p* < 0.05, Figure 5). The 500-mers exhibit a greater range of decay rates, with an average rate of 0.12 ± 0.015 min^–1^ for the *trans*-enriched polymers, 0.37 ± 0.099 min^–1^ for the polymers with an intermediate composition, and 0.53 ± 0.13 min^–1^ for the *cis*-enriched polymers. Even accounting for individual variation in exact *M*_n_ and dispersity, both the intermediate and *cis*-enriched polymers have a significantly faster degradation rate than the *trans*-enriched polymers (*p* < 0.05, Figure 5).

**Figure 5:**
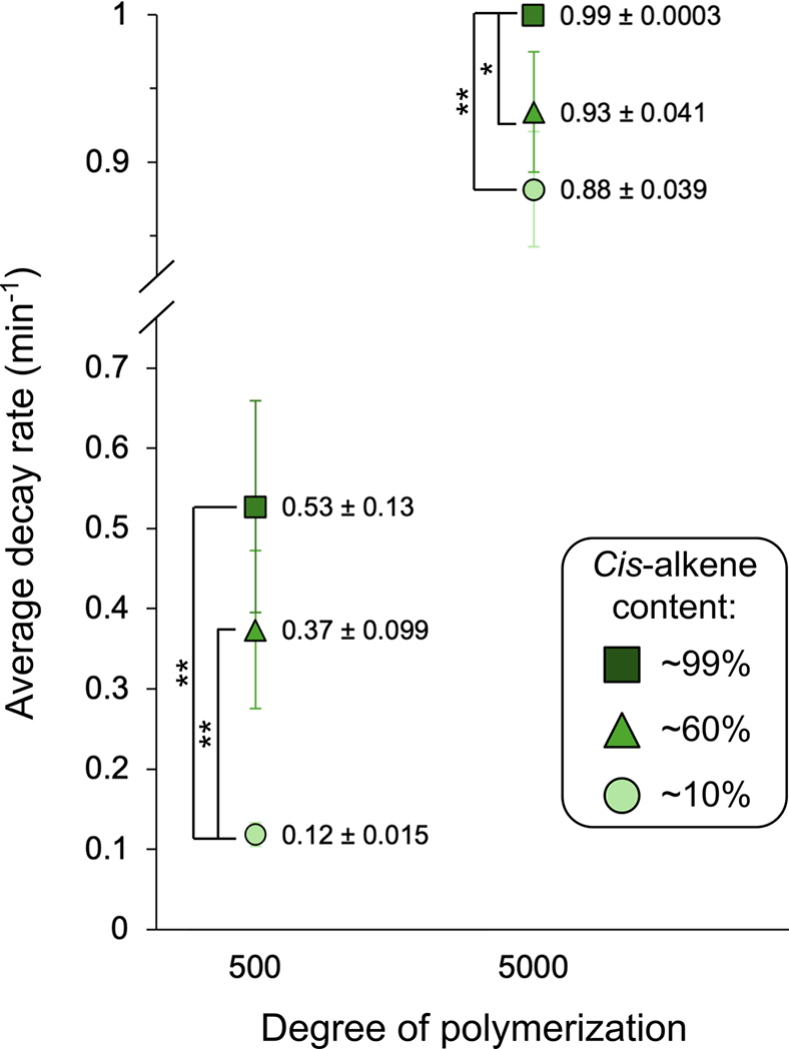
Average polynorbornene ultrasonic degradation rates as a function of degree of polymerization and *cis*-alkene content. Degradation rates were extracted from exponential decay fits to the *M*_n_ values over 6 h of ultrasonic degradation. Data are presented as the average of triplicate measurements from three distinct samples (a–c of each numbered entry in Table 1) with error bars representing the standard deviation of the mean. Statistical analysis was performed with Welch’s *t* test of paired samples: * *p* ≤ 0.1, ** *p* < 0.05.

The corresponding *M*_w_ analysis revealed an identical trend (Figure S76). The higher variability in decay rate for the 500-mers with intermediate and high *cis*-alkene content can likely be ascribed to small differences in dispersity and the outsized effect that a high-molecular weight shoulder has on these slower-degrading materials. Still, all 500-mers degraded slower than their 5000-mer counterparts and exhibited a similar increase in degradation rate with increasing *cis*-alkene content. While the effect of DP on scission rate is well-understood, the effect of backbone stereochemistry on scission rate is previously unreported and offers an exciting new handle for tuning material degradability.

## Conclusions

Polynorbornenes of both ∼700 and ∼70 kDa with a backbone composition ranging from ∼10% to ∼99% *cis*-alkenes were synthesized by ROMP and then degraded by ultrasonication. We found that highly *cis*-enriched polynorbornenes degraded faster (with decay rates up to 0.999 min^–1^) than their *trans*-enriched analogues at similar molecular weights and dispersities, as determined by GPC. These results are consistent with literature precedent that mechanical degradability increases with polymer stiffness and therefore suggest that increasing the *cis*-alkene content in polynorbornene may promote a more rigid, elongated structure. Efforts to theoretically model and experimentally quantify the shape of these polymers and other polyalkenamers with variable allylic strain in their backbones are ongoing. Our findings indicate that the stereocontrolled synthesis of polyalkenamers can drive susceptibility to mechanical degradation, which will be important for the design of recyclable plastics.

## Supplementary Material



## Notes

Synthetic and experimental protocols, ^1^H NMR spectra, GPC chromatograms, degradation rates, and additional tables and figures (PDF)

## †

Cornell University, Ithaca, New York 14853, United States

## ‡

Colorado State University, Ft. Collins, Colorado 80523, United States
