# Supplementary material for: Impact of Stereocontrolled Polynorbornene Synthesis on Degradation Rate
Source: Macromolecules. 2025 Aug 29;58(18):10094–101. doi: 10.1021/acs.macromol.5c01049 (PMC12461934; doi:10.1021/acs.macromol.5c01049)
Supplement: Supplementary file 1 [file ma5c01049_si_001.pdf]

Supporting Information for

Impact of Stereocontrolled Polynorbornene Synthesis on Degradation Rate

Britney Baez, Greyson Karis-Sconyers, Ethan Flanagan, Aech Loar, and Spencer D. Brucks*

Department of Chemistry, Harvey Mudd College, Claremont, California, United States

*Corresponding author: sbrucks@.hmc.edu

Table of Contents

1. Materials 2

2. Abbreviations 2

3. Instrumentation...3

4. Experimental Methods 3

4.1 Polynorbornene Synthesis...3

4.2 Ultrasonication Degradation Experiments...............................6

4.3 GPC Data Processing and Analysis 6

5. Supplemental Tables and Figures 11

5.1 Supplemental Data Tables 11

5.2 NMR Spectra of Undegraded Polymers 16

5.3 Overlaid GPC Chromatograms over Six Hour Degradation.............20

5.4 Scission Cycle Analysis...27

5.5 Ultrasonication Degradation Controls 34

5.6 Initial Peak Molecular Weight Intensity Analysis 39

5.7 Degradation Rate Analysis 46

6. References 56

1. Materials

Dichloromethane, Benzylidene-bis(tricyclohexylphosphine)dichlororuthenium (Grubbs 1st generation catalyst), (1,3-Bis(2,4,6-trimethylphenyl)-2-imidazolidinylidene)dichloro(phenylmethylene)(tricyclohexylphosphine) ruthenium (Grubbs 2nd generation catalyst), [2-(1-Methylethoxy-*O*)phenylmethyl-*C*](nitrato-*O,O'*){*rel*-(2*R*,5*R*,7*R*)adamantane-2,1-diyl[3-(2,4,6-trimethylphenyl)-1-imidazolidinyl-2-ylidene]}ruthenium(Hoveyda-Grubbs M2001 catalyst), 3-bromopyridine, ethyl vinyl ether, and chloroform-d ($CDCl_3$), were all purchased from MilliporeSigma (Burlington, MA). Norbornene was purchased from TCI Chemicals (Portland, OR). Methanol and butylated hydroxytoluene were purchased from Thermo Fisher Scientific (Waltham, MA). 3,5-dichloropyridine was purchased from Santa Cruz Biotechnology (Dallas, TX).

Grubbs 3rd generation catalyst was prepared from Grubbs 2nd generation catalyst as reported in the literature.[1]

Tetrahydrofuran was obtained under a nitrogen atmosphere from an anhydrous solvent distillation system, PS-400-5MD (Innovative Technology Inc., Newburyport, MA).

M3002 catalyst was kindly donated by Umicore.

All glassware and stir bars were dried in an oven at 150 ºC prior to use.

All reactions were carried out under an inert atmosphere of N_2 using standard Schlenk technique.

2. Abbreviations

BHT	Butylated hydroxytoluene
Cl_2Py	3,5-dichloropyridine
DCM	Dichloromethane
G1	Grubbs 1st generation catalyst
G2	Grubbs 2nd generation catalyst
G3	Grubbs 3rd generation catalyst
GPC	Gel permeation chromatography
HG M2001	Hoveyda-Grubbs M2001 catalyst
M3002	Grubbs M3002 catalyst
ROMP	Ring-opening metathesis polymerization
THF	Tetrahydrofuran

3. Instrumentation

All NMR spectra were obtained with a Bruker AvanceNEO 400 MHz spectrometer and referenced to residual solvent peaks.

Gel permeation chromatography was performed on a Tosoh HLC-8420 EcoSEC Elite equipped with a TSKgel GMH_{HR}-M column packed with 5 μM beads using a mobile phase of tetrahydrofuran with 250 ppm BHT and a flow rate of 1 mL/min at 40 °C. Molecular weights were determined by referencing a calibration curve prepared from polystyrene standards and RI detection.

Ultrasonication experiments were conducted in a FisherBrand FB11203 Advanced Ultrasonic Cleaner at 37 kHz and ~0.4 W cm^{-2} with the water bath held at 26 °C using a Cole Parmer 1C6 Polystat recirculating water chiller.

Polymerization of norbornene with Grubbs M3002 catalyst was conducted in a MBraun UNIlab glovebox.

4. Experimental Methods

4.1 Polynorbornene Synthesis

G1 5000-mer: To an oven-dried 20 mL scintillation vial equipped with a stir bar, 85 mg (0.9 mmol) of norbornene was added and dissolved in 8.6 mL (~0.105 M) anhydrous THF. A G1 stock solution was then prepared in a separate oven-dried vial wrapped in foil by dissolving the catalyst in anhydrous THF at a concentration of 0.3 mg/mL. The norbornene solution was placed under N_2 at 40 °C and stirring was turned on to 800 rpm. Next, 0.5 mL (~0.00018 mmol catalyst) of the catalyst solution was rapidly injected via syringe for a final concentration of ~0.1 M norbornene. The vial was stirred at 40 °C for 10 minutes, then quenched with excess ethyl vinyl ether. The polymer solution was diluted with additional THF then precipitated into ice-cold methanol, and isolated by vacuum filtration. A representative ^{1}H NMR in $CDCl_3$ is presented as Figure S2.

G1 500-mer: To an oven-dried 20 mL scintillation vial equipped with a stir bar, 85 mg (0.9 mmol) of norbornene was added and dissolved in 8.1 mL (0.11 M) anhydrous THF. A G1 stock solution was then prepared in a separate oven-dried vial wrapped in foil by dissolving the catalyst in anhydrous THF at a concentration of 1.5 mg/mL. The norbornene solution was placed under N_2 at 40 °C and stirring was turned on to 800 rpm. Next, 1 mL (~0.0018 mmol catalyst) of the catalyst solution was rapidly injected via syringe for a final concentration of ~0.1 M norbornene. The vial was stirred at 40 °C for 10 minutes, then quenched with excess ethyl vinyl ether. The polymer solution was precipitated into ice-cold methanol, and isolated by vacuum filtration. A representative ^{1}H NMR in $CDCl_3$ is presented as Figure S5.

G1 200-mer: To an oven-dried 20 mL scintillation vial equipped with a stir bar, 80 mg (0.85 mmol) of norbornene was added and dissolved in 1.9 mL (0.45 M) anhydrous THF. A G1 stock

solution was then prepared in a separate oven-dried vial by dissolving the catalyst in anhydrous THF at a concentration of 3.9 mg/mL. The norbornene solution was placed under N_2 at 40 °C and stirring was turned on to 800 rpm. Next, 0.9 mL (~0.0043 mmol catalyst) of the catalyst solution was rapidly injected via syringe for a final concentration of ~0.3 M norbornene. The vial was stirred at 40 °C for 10 minutes, then quenched with excess ethyl vinyl ether. The polymer solution was precipitated into ice-cold methanol, and isolated by vacuum filtration. A ^{1}H NMR was collected and analyzed similar to other polymers and had a *cis*-alkene content of 12 %.

G3 5000-mer: To an oven-dried 20 mL scintillation vial equipped with a stir bar, 160 mg (1.7 mmol) of norbornene was added and dissolved in 4.7 mL (0.36 M) DCM. A G3 stock solution was then prepared in a separate oven-dried vial by dissolving the catalyst in DCM at a concentration of 0.25 mg/mL. The norbornene solution was placed under N_2 in a salt-water ice bath and stirring was turned on to 800 rpm. Next, 1 mL (~0.0003 mmol catalyst) of the catalyst solution was rapidly injected via syringe for a final concentration of ~0.3 M norbornene. The reaction was stirred on the ice bath for 30 minutes, then quenched with excess ethyl vinyl ether. The polymer solution was diluted with additional THF then precipitated into ice-cold methanol and isolated by vacuum filtration. A representative ^{1}H NMR in $CDCl_3$ is presented as Figure S3.

G3 500-mer: To an oven-dried 20 mL scintillation vial equipped with a stir bar, 160 mg (1.7 mmol) of norbornene was added and dissolved in 4.7 mL (0.36 M) DCM. A G3 stock solution was then prepared in a separate oven-dried vial by dissolving the catalyst in DCM at a concentration of 3.0 mg/mL. The norbornene solution was placed under N_2 in a salt-water ice bath and stirring was turned on to 800 rpm. Next, 1 mL (~0.0034 mmol catalyst) of the catalyst solution was rapidly injected via syringe for a final concentration of ~0.3 M norbornene. The reaction was stirred on the ice bath for 15 minutes, then quenched with excess ethyl vinyl ether. The polymer solution was precipitated into ice-cold methanol and isolated by vacuum filtration. A representative ^{1}H NMR in $CDCl_3$ is presented as Figure S6.

G3 200-mer: To an oven-dried 20 mL scintillation vial equipped with a stir bar, 64 mg (0.68 mmol) of norbornene was added and dissolved in 1.5 mL (0.45 M) anhydrous THF. A G3 stock solution was then prepared in a separate oven-dried vial by dissolving the catalyst in anhydrous THF at a concentration of 3.75 mg/mL. The norbornene solution was placed under N_2 in a salt-water ice bath and stirring was turned on to 800 rpm. Next, 0.8 mL (~0.0034 mmol catalyst) of the catalyst solution was rapidly injected via syringe for a final concentration of ~0.3 M norbornene. The reaction was stirred on the ice bath for 15 minutes, then quenched with excess ethyl vinyl ether. The polymer solution was precipitated into ice-cold methanol and isolated by vacuum filtration. A ^{1}H NMR was collected and analyzed similar to other polymers and had a *cis*-alkene content of 61%.

HG M2001 5000-mer: To an oven-dried 20 mL scintillation vial equipped with a stir bar, 150 mg (1.6 mmol) of norbornene was added and dissolved in 4.3 mL (0.37 M) DCM. A HG stock solution was then prepared in a separate oven-dried vial by dissolving the catalyst in DCM at a concentration of 2.0 mg/mL. The norbornene solution was placed under N_2 in a dry ice / acetone bath and stirring was turned on to 800 rpm. Next, 1 mL (~0.003 mmol catalyst) of the catalyst solution was rapidly injected via syringe for a final concentration of 0.3 M norbornene and a monomer:catalyst ratio of 500:1. The reaction was stirred on the dry ice / acetone bath for 20

minutes, then quenched with excess ethyl vinyl ether. The polymer solution was precipitated into ice-cold methanol and isolated by vacuum filtration. A representative ^{1}H NMR in $CDCl_3$ is presented as Figure S4.

M3002 500-mer: In a glovebox, 160 mg (1.7 mmol) of norbornene was added to a 20 mL scintillation vial equipped with a stir bar and dissolved in 4.7 mL (0.36 M) anhydrous DCM. An M3002 catalyst stock solution was then prepared in a separate vial by dissolving the catalyst in anhydrous DCM at a concentration of 3.0 mg/mL with an additional 6.5 mg/mL of Cl_2Py dissolved in the same solution as a stabilizer. Stirring was initiated for the norbornene solution and then 1 mL (0.0035 mmol catalyst, 0.045 mmol Cl_2Py) of the stabilized catalyst solution was rapidly injected via syringe for a final concentration of ~0.3 M norbornene. The reaction was stirred at room temperature for 10 minutes, then quenched with excess ethyl vinyl ether. The quenched reaction was then removed from the glovebox, concentrated by solvent evaporation, and finally precipitated into ice-cold methanol and isolated by vacuum filtration. A representative ^{1}H NMR in $CDCl_3$ is presented as Figure S7.

M3002 300-mer: In a glovebox, 114 mg (1.21 mmol) of norbornene was added to a 20 mL scintillation vial equipped with a stir bar and dissolved in 3.0 mL (0.40 M) anhydrous THF. An M3002 catalyst stock solution was then prepared in a separate vial by dissolving the catalyst in anhydrous THF at a concentration of 3.5 mg/mL with an additional 9 mg/mL of Cl_2Py dissolved in the same solution as a stabilizer. Stirring was initiated for the norbornene solution and then 1 mL (0.004 mmol catalyst, 0.06 mmol Cl_2Py) of the stabilized catalyst solution was rapidly injected via syringe for a final concentration of ~0.3 M norbornene. The reaction was stirred at room temperature for 10 minutes, then removed from the glovebox and quenched with excess ethyl vinyl ether. The reaction was then concentrated by solvent evaporation, and finally precipitated into ice-cold methanol and isolated by vacuum filtration. ^{1}H NMR was collected and analyzed similar to other polymers and had a *cis*-alkene content of 100 %.

M3002 200-mer: In a glovebox, 76 mg (0.81 mmol) of norbornene was added to a 20 mL scintillation vial equipped with a stir bar and dissolved in 1.7 mL (0.48 M) anhydrous THF. An M3002 catalyst stock solution was then prepared in a separate vial by dissolving the catalyst in anhydrous THF at a concentration of 3.5 mg/mL with an additional 4.5 mg/mL of Cl_2Py dissolved in the same solution as a stabilizer. Stirring was initiated for the norbornene solution and then 1 mL (0.004 mmol catalyst, 0.03 mmol Cl_2Py) of the stabilized catalyst solution was rapidly injected via syringe for a final concentration of ~0.3 M norbornene. The reaction was stirred at room temperature for 10 minutes, then removed from the glovebox and quenched with excess ethyl vinyl ether. The reaction was then concentrated by solvent evaporation, and finally precipitated into ice-cold methanol and isolated by vacuum filtration. ^{1}H NMR was collected and analyzed similar to other polymers and had a *cis*-alkene content of 100 %.

4.2 Ultrasonication Degradation Experiments

For all sonication experiments, 20 mg of a polymer sample was dissolved in 20 mL of THF stabilized with 250 ppm BHT in a 20 mL scintillation vial. High-molecular weight samples were solubilized overnight to allow for complete dissolution. The vial was localized to the center of the sonication bath using a floating foam tube rack, ensuring the entire sample volume was fully submerged below the water line. The ultrasonicator was set at 37 kHz and ~0.4 W cm^{-2} with the water bath held constant at 26 ºC. Ultrasonication proceeded for six cumulative hours with aliquots taken according to one of the following schedules:

Low molecular weight samples

5 min	45 min	180 min
10 min	60 min	240 min
15 min	90 min	300 min
30 min	135 min	360 min

High molecular weight samples

1 min	25 min	180 min
3 min	45 min	240 min
5 min	90 min	300 min
10 min	135 min	360 min

All aliquots were filtered through a 0.22 µM 13 mm diameter PTFE filter and then characterized by gel permeation chromatography on the same day as their collection.

4.3 GPC Data Processing and Analysis

All GPC curves were processed with a baseline correction which subtracted the slope between the ~5 min and ~9 min elution time points with no sample elution and then zeroed at the ~5 min mark. The processed data were imported to Visual Studio Code as a CSV file and then normalized by peak area and visualized using the Python script below (printed for analysis of one polymer data set collected on the small molecular weight schedule).

We analyzed the change in initial M_p intensity over sonication time by measuring the refractive index (RI) signal at the GPC retention time corresponding to the M_p of the parent polymer across all degradation aliquots. The RI values were then normalized twice: first by peak area to account for concentration differences across samples, and then by the parent M_p intensity to ensure that the initial RI of the M_p was equal to 1.0 at t = 0 min for all samples.

Plots of M_w and M_n versus sonication time were made and fit to the exponential decay model $Y = A(1 - r)^t + c$, where Y is the M_w (or M_n) after degradation time t, r is the decay rate, c is a constant, and A is a pre-exponential term. Each of these fitting parameters and the R^2 value of every fit was extracted in Python.

Representative Python Script

```
import numpy as np
import scipy
import scipy.optimize
import scipy.stats
import matplotlib.pyplot as plt
import matplotlib as mpl
import csv
import math

# read in data from a CSV file
f = open('42-corrected.csv')
reader = csv.reader(f)
P1_raw = []
for row in reader:
    P1_raw.append(row)
P1_raw = P1_raw[4:]
P1_times = [float(x[0]) for x in P1_raw]
P1_0 = [float(x[1]) for x in P1_raw]
P1_5 = [float(x[3]) for x in P1_raw]
P1_10 = [float(x[5]) for x in P1_raw]
P1_15 = [float(x[7]) for x in P1_raw]
P1_30 = [float(x[9]) for x in P1_raw]
P1_45 = [float(x[11]) for x in P1_raw]
P1_60 = [float(x[13]) for x in P1_raw]
P1_90 = [float(x[15]) for x in P1_raw]
P1_135 = [float(x[17]) for x in P1_raw]
P1_180 = [float(x[19]) for x in P1_raw]
P1_240 = [float(x[21]) for x in P1_raw]
P1_300 = [float(x[23]) for x in P1_raw]
P1_360 = [float(x[25]) for x in P1_raw]
P1_data = [P1_0, P1_5, P1_10, P1_15, P1_30, P1_45, P1_60, P1_90, P1_135, P1_180,
P1_240, P1_300, P1_360]
# time points at which data was collected
times = [0, 5, 10, 15, 30, 45, 60, 90, 135, 180, 240, 300, 360]
times = [x / 60 for x in times] # convert to hours
#
# some useful functions
#
def time_to_mw(t):
    # converts times to molecular weights based on
    # calibration curve
    return 10 ** (-0.8736920591*t + 10.93438581)
# convert times to molecular weights for our data
P1_mws = [time_to_mw(t) for t in P1_times]
def mw3(signal, weight):
    # calculate Mw based on GPC response ('signal' vector)
```

```
    # and corresponding molecular weights ('weight' vector).
    # Data passed in should only be for the peak for which Mw
    # is being calculated.
    integral = np.float64(sum([signal[i] for i in range(len(signal))]))
    return sum([signal[i]/integral * weight[i] for i in range(len(signal))])
def mn3(signal, weight):
    # calculate Mn based on GPC response ('signal' vector) and corresponding
    # molecular weights ('weight' vector).
    # data passed in should only be for the peak for which Mn is being calculated.
    integral = np.float64(sum([signal[i] for i in range(len(signal))]))
    return integral / sum([signal[i] / weight[i] for i in range(len(signal))])
#
# functions to fit Mn vs t and Mw vs t data to
#
def mn_fit(ts, *params):
    result = []
    a, r, c = params
    for t in ts:
        result.append((a * (1 - r)**t) + c)
    return result

def mw_fit(ts, *params):
    result = []
    a, r, c = params
    for t in ts:
        result.append((a * (1 - r)**t) + c)
    return result
#
# actual plotting code
#
# custom colormap for times -> colors
cdict = {'red': [[0.0, 1.0, 1.0],
                 [0.2, 0.5, 0.5],
                 [1.0, 0.0, 0.0]],
       'green': [[0.0, 0.25, 0.25],
                 [0.1, 0.5, 0.5],
                 [1.0, 0.8, 0.8]],
        'blue': [[0.0, 0.0, 0.0],
                 [0.1, 0.5, 0.5],
                 [0.4, 1.0, 1.0],
                 [1.0, 0.7, 0.7]]}
newcmp = mpl.colors.LinearSegmentedColormap('testCmap',
                                            segmentdata=cdict, N=256)
cmap = newcmp
norm = mpl.colors.Normalize(vmin=0, vmax=max(times))
# set up subplot layout
gs_kw = dict(width_ratios=[3, 1], height_ratios=[1, 1])
fig, axd = plt.subplot_mosaic([['left', 'upper right'],
```

```
                              ['left', 'lower right']],
                              gridspec_kw=gs_kw, figsize=(10, 5),
                              layout="constrained", dpi=100)

plotmws =
[710666,451187,388924,323638,207070,160542,139323,98335,63798,50984,42240,40680,31135]
plotmns =
[514933,313535,267084,224551,147943,116255,101529,74550,49005,39309,32976,31762,24369]
# loop through dataset
for i in range(len(P1_data)):
    d = P1_data[i]
    # calculate integral in order to normalize
    integral = np.trapz(d[2800:5200], P1_times[2800:5200])
    # plot normalized GPC chromatogram on left axis
    axd['left'].plot(P1_mws[2800:5500],
                 [d[j]/integral for j in range(2800,5500)],
                 color=cmap(norm(times[i])))
axd['left'].set_xscale('log')
axd['left'].set_xlabel('Molecular Weight / Da')
axd['left'].set_ylabel('dw / dLog(M)')
# fit Mn and Mw curves to functions
fit1, pcov = scipy.optimize.curve_fit(mn_fit,times,plotmns,p0=(10,0.5,10),maxfev=5000)
fit2,_ = scipy.optimize.curve_fit(mw_fit,times,plotmws,p0=(10,0.5,10),maxfev=5000)

# calculate R^2 for the Mn fit
def Mn_res(mns,ts,*fit):
    result = []
    for i in range(len(mns)):
        result.append(mns[i] - (mn_fit(ts,*fit))[i])
    return result

def Mn_SSres(resL):
    myL = []
    for res in resL:
        myL.append(res**2)
    return np.sum(myL)

def Mn_SStot(mns):
    myL = []
    for mw in mns:
        myL.append((mw - np.mean(mns))**2)
    return np.sum(myL)

Mn_r_squared = 1 - (Mn_SSres(Mn_res(plotmns,times,*fit1)) / Mn_SStot(plotmns))
# print(Mn_r_squared)

# calculate R^2 for the Mw fit
def Mw_res(mws,ts,*fit):
```

```
    result = []
    for i in range(len(mws)):
        result.append(mws[i] - (mw_fit(ts,*fit))[i])
    return result

def Mw_SSres(resL):
    myL = []
    for res in resL:
        myL.append(res**2)
    return np.sum(myL)

def Mw_SStot(mws):
    myL = []
    for mw in mws:
        myL.append((mw - np.mean(mws))**2)
    return np.sum(myL)

Mw_r_squared = 1 - (Mw_SSres(Mw_res(plotmws,times,*fit2)) / Mw_SStot(plotmws))

axd['upper right'].plot(times, plotmns, 'o')
axd['upper right'].plot(np.linspace(0, max(times), 150),
                        mn_fit(np.linspace(0, max(times), 150), *fit1))
axd['lower right'].plot(times, plotmws, 'o')
axd['lower right'].plot(np.linspace(0, max(times), 150),
                        mw_fit(np.linspace(0, max(times), 150), *fit2))
axd['upper right'].set_xlabel('Time / hr')
axd['lower right'].set_xlabel('Time / hr')
axd['upper right'].set_ylabel('Mn')
axd['lower right'].set_ylabel('Mw')
# plot colorbar
fig.colorbar(mpl.cm.ScalarMappable(norm=norm, cmap=cmap),
             ax=axd['left'], orientation='vertical', label='Time / hr')
# give figure a title
fig.suptitle('42, 6 hours degradation')
plt.show()
```

5. Supplemental Tables and Figures

5.1 Supplemental Data Tables

Entry	Catalyst	Monomer:catalyst ratio	Mn (kDa)[a]	Mw (kDa)[a]	Đ[a]	Cis %[b]	Condition
1a	G3	500	93	100	1.08	61	dry ice/acetone
1b	G3	500	37	40	1.08	62	dry ice/acetone
1c	G3	500	72	89	1.25	60	dry ice/acetone
1d	G3	500	62	85	1.38	60	dry ice/acetone
1e	G3	500	50	60	1.09	61	salt + ice bath
1f	G3	500	65	75	1.16	61	salt + ice bath
1g	G3	500	53	69	1.30	60	salt + ice bath
1h	G3	500	49	60	1.22	61	salt + ice bath
1i	G3	500	60	70	1.22	61	ice
1j	G3	500	57	104	1.81	60	ice
1k	G3	500	41	63	1.51	61	ice
1l	G3	500	41	64	1.57	61	ice
1m	G3	500	50	106	2.10	59	rt
1n	G3	500	45	87	1.92	59	rt
1o	G3	500	40	82	2.06	59	rt
1p	G3	500	38	97	2.57	59	rt
1q	G3	500	25	79	3.10	58	35 °C
1r	G3	500	28	79	2.88	58	35 °C
1s	G3	500	27	87	3.24	58	35 °C
1t	G3	500	25	82	3.22	58	35 °C
2a	HG	50	230	530	2.33	96	salt + ice bath
2b	HG	50	160	360	2.26	96	salt + ice bath
2c	HG	500	180	400	2.10	96	salt + ice bath
2d	HG	500	175	310	1.77	96	salt + ice bath
2e	HG	500	400	615	1.55	98	dry ice/acetone
2f	HG	500	190	310	1.66	98	dry ice/acetone
2g	HG	500	580	885	1.53	100	dry ice/acetone
2h	HG	500	560	750	1.35	100	dry ice/acetone
3a	G1	500	35	110	3.31	n.d.	salt + ice bath
3b	G1	500	10	30	2.74	n.d.	salt + ice bath
3c	G1	500	70	170	2.43	n.d.	salt + ice bath
3d	G1	500	45	125	2.86	n.d.	salt + ice bath
3e	G1	500	45	120	2.77	n.d.	salt + ice bath
3f	G1	500	85	170	2.08	n.d.	salt + ice bath
3g	G1	500	40	100	2.47	n.d.	35 °C DCM
3h	G1	500	50	90	1.80	n.d.	50 °C THF
3i	G1	500	55	150	2.81	n.d.	0.1 M rt DCM
3j	G1	500	65	225	3.41	n.d.	0.3 M rt DCM
3k	G1	500	45	85	1.87	n.d.	0.1 M 50 °C THF
3l	G1	500	50	100	1.93	n.d.	0.3 M 50 °C THF
3m	G1	500	60	75	1.27	11	0.1 M 5 min THF 40 °C
3n	G1	500	60	78	1.36	12	0.1 M 5 min THF 60 °C
3o	G1	500	40	70	1.64	n.d.	0.1 M 5 min THF rt
3p	G1	500	50	70	1.39	n.d.	0.1 M 5 min THF 30 °C
3q	G1	500	65	80	1.28	11	0.1 M 5 min THF 40 °C
3r	G1	5000	445	685	1.54	n.d.	0.1 M 5 min THF 40 °C

[caption refers to table on previous page]
Table S1. Reaction condition optimization testing for each ruthenium-catalyzed polymerization. [a]Determined by size exclusion chromatography in THF using polystyrene standards and RI detection. [b]Determined by ^1H NMR (400 MHz, $CDCl_3$). N.d. represents value not determined.

Time (min)	Mn	Mw	Đ
0	554193	886178	1.60
1	548457	880485	1.61
3	546972	893440	1.63
5	491973	770381	1.57
10	456901	691099	1.51
25	283967	409907	1.44
45	189276	265634	1.40
90	141320	194393	1.38
135	113571	152414	1.34
180	101435	133575	1.32
240	83930	110024	1.31
300	65557	84779	1.29
360	53087	68983	1.30

Table S2. Molecular weight characteristics of every aliquot for **Table 1** entry **1c** (**Figure 3A** top) taken across six cumulative hours of ultrasonication.

Time (min)	Mn	Mw	Đ
0	54332	73697	1.36
5	53139	75632	1.42
10	50986	75641	1.48
15	51212	75431	1.47
30	50891	71662	1.41
45	49998	70701	1.41
60	50770	69987	1.38
90	48649	68265	1.40
135	49230	66244	1.35
180	48992	65598	1.34
240	45435	61576	1.36
300	45423	60703	1.34
360	43560	59139	1.36

Table S3. Molecular weight characteristics of every aliquot for **Table 1** entry **2b** (**Figure 3B** top) taken across six cumulative hours of ultrasonication.

Time (min)	Mn	Mw	Đ
0	514933	710666	1.38
5	313535	451187	1.44
10	267084	388924	1.46
15	224551	323638	1.44
30	147943	207070	1.40
45	116255	160542	1.38
60	101529	139323	1.37
90	74550	98335	1.32
135	49005	63798	1.30
180	39309	50984	1.30
240	32976	42240	1.28
300	31762	40680	1.28
360	24369	31135	1.28

Table S4. Molecular weight characteristics of every aliquot for **Table 1** entry **3c** (**Figure 3A** middle) taken across six cumulative hours of ultrasonication. *Note: This high molecular weight polymer was ultrasonicated according to the "low molecular weight" schedule.*

Time (min)	Mn	Mw	Đ
0	58081	70915	1.22
5	57056	69879	1.23
10	56402	68948	1.22
15	54960	68044	1.24
30	51883	64853	1.25
45	48482	60913	1.26
60	46445	59103	1.27
90	43310	56354	1.30
135	39714	52985	1.33
180	38465	50807	1.32
240	37135	48982	1.32
300	35104	46883	1.34
360	33551	44865	1.34

Table S5. Molecular weight characteristics of every aliquot for **Table 1** entry **4c** (**Figure 3B** middle) taken across six cumulative hours of ultrasonication.

Time (min)	Mn	Mw	Đ
0	434253	684370	1.58
1	297472	564389	1.90
3	214477	399827	1.86
5	165257	301321	1.82
10	135474	231766	1.71
25	52692	75058	1.42
45	37274	49346	1.32
90	29096	37761	1.30
135	23950	30231	1.26
180	21611	26954	1.25
240	18334	23599	1.29
300	18493	22715	1.23
360	17279	21055	1.22

Table S6. Molecular weight characteristics of every aliquot for **Table 1** entry **5a** (**Figure 3A** bottom) taken across six cumulative hours of ultrasonication.

Time (min)	Mn	Mw	Đ
0	43395	58242	1.34
5	41341	54155	1.31
10	41085	53786	1.31
15	40590	52805	1.30
30	36339	47057	1.30
45	34588	43945	1.27
60	32211	41145	1.28
90	30292	38199	1.26
135	28242	35649	1.26
180	23221	29205	1.26
240	21297	26479	1.24
300	20174	24947	1.24
360	19982	24633	1.23

Table S7. Molecular weight characteristics of every aliquot for **Table 1** entry **6b** (**Figure 3B** bottom) taken across six cumulative hours of ultrasonication.

Entry	*Cis*-alkenes (%)	Mn	Mw
1	12	22546	28401
2	61	16440	20549
3	100	12625	15247

Table S8. Molecular weight characteristics of 500-mer polynorbornenes after 24 hours of continuous ultrasonication in THF at 26 °C.

Entry	Catalyst	M/I/Cl_2Py	Condition	Yield (%)	M_n (kDa)	M_w (kDa)	Đ	*Cis*-alkenes (%)
**7**	G1	200/1/0	40 ºC, 0.3 M THF, 10 mins	70	20.5	31.2	1.53	12
**8**	G3	200/1/0	-20 ºC, 0.3 M THF, 15 mins	53	20.5	22.2	1.08	61
**9**	M3002	300/1/15	20 ºC, 0.3 M THF, 10 mins	40	25.5	27.7	1.08	100
**10**	M3002	200/1/7.5	20 ºC, 0.3 M THF, 10 mins	50	11.4	12.6	1.11	100

Table S9. Library of synthesized polynorbornenes at molecular weight limit of degradation.

Time (hr)	Mn	Mw
0	434253	684370
24	370471	681772

Table S10. Molecular weight characteristics of a 99% *cis* polynorbornene **5a** after 0 and 24 hours of dissolution in THF without ultrasonication.

5.2 NMR Spectra of Undegraded Polymers

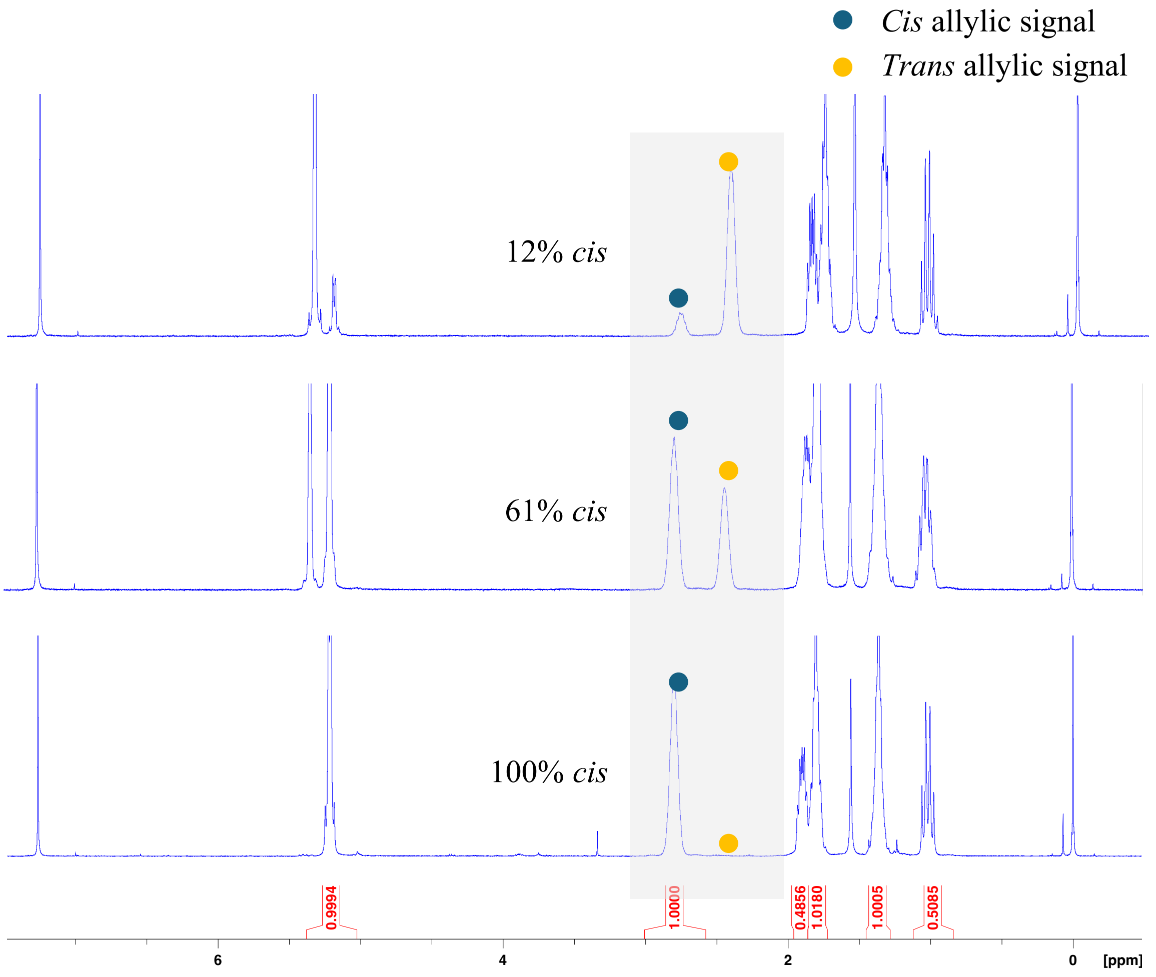

Figure S1. Quantification of *cis/trans* alkene ratio in polynorbornene via ^{1}H NMR quantification of allylic hydrogen signal.

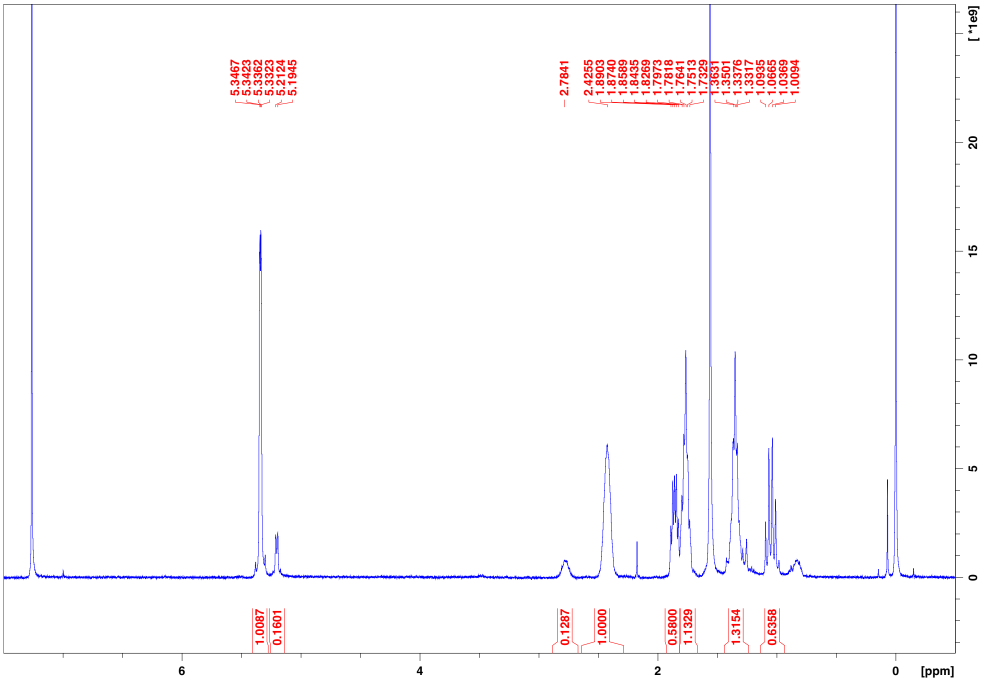

Figure S2. ^{1}H NMR (400 MHz, $CDCl_3$) spectrum of **Table 1** entry **1c** with ~10% *cis*-alkenes in polymer backbone.

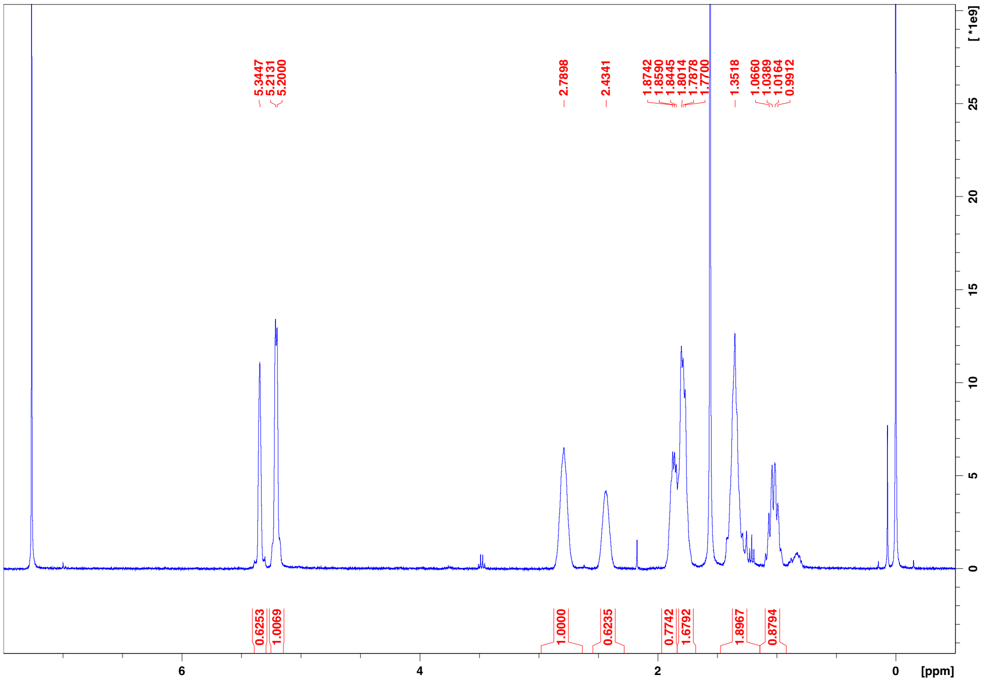

Figure S3. ^{1}H NMR (400 MHz, $CDCl_3$) spectrum of **Table 1** entry **3c** ~60% *cis*-alkenes in polymer backbone.

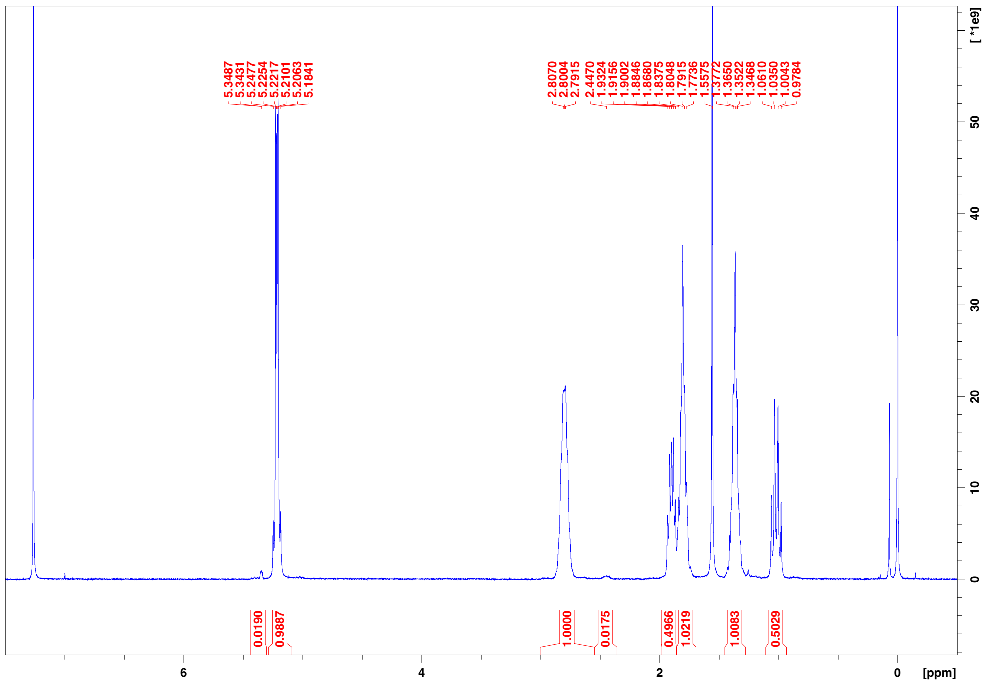

Figure S4. ^{1}H NMR (400 MHz, $CDCl_3$) spectrum of **Table 1** entry **5a** with 99% *cis*-alkenes in polymer backbone.

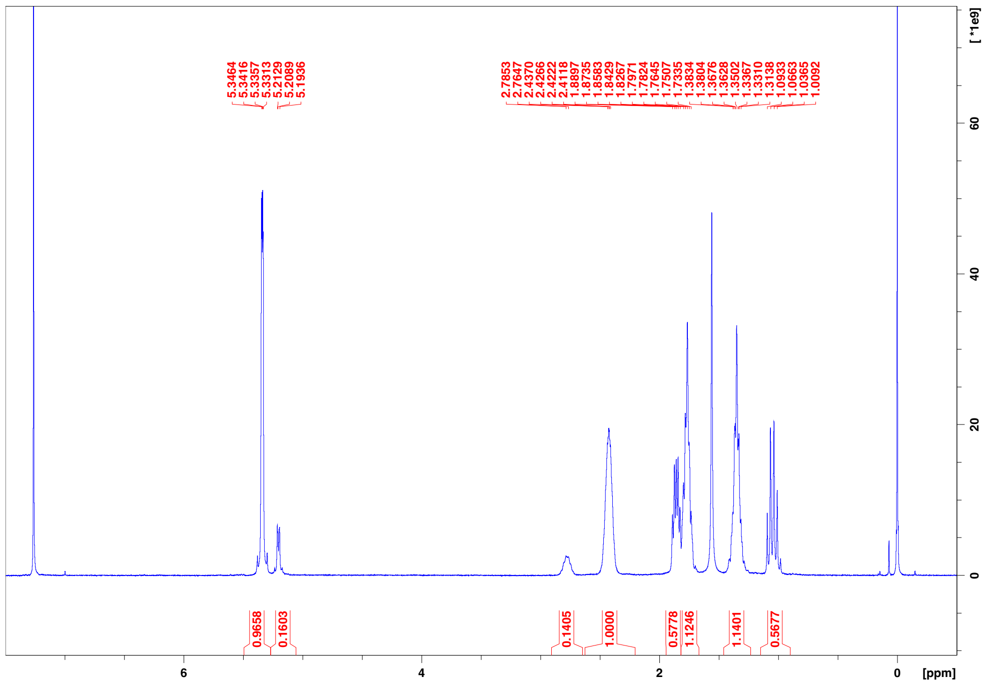

Figure S5. ^{1}H NMR (400 MHz, $CDCl_3$) spectrum of **Table 1** entry **2b** ~10% *cis*-alkenes in polymer backbone.

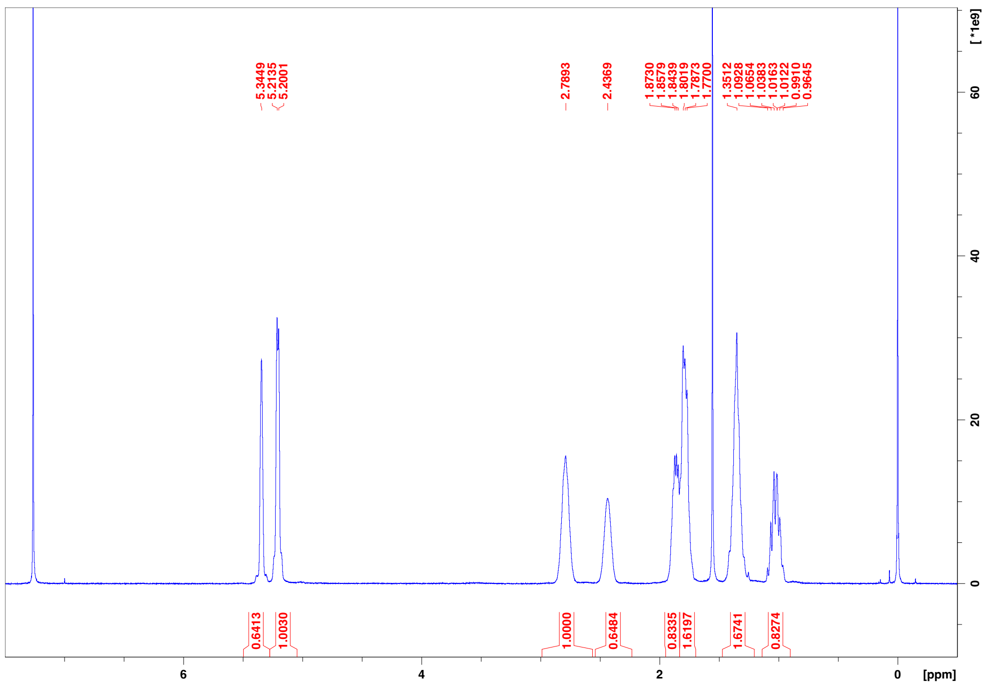

Figure S6. ^{1}H NMR (400 MHz, $CDCl_3$) spectrum of **Table 1** entry **4c** with ~60% *cis*-alkenes in polymer backbone.

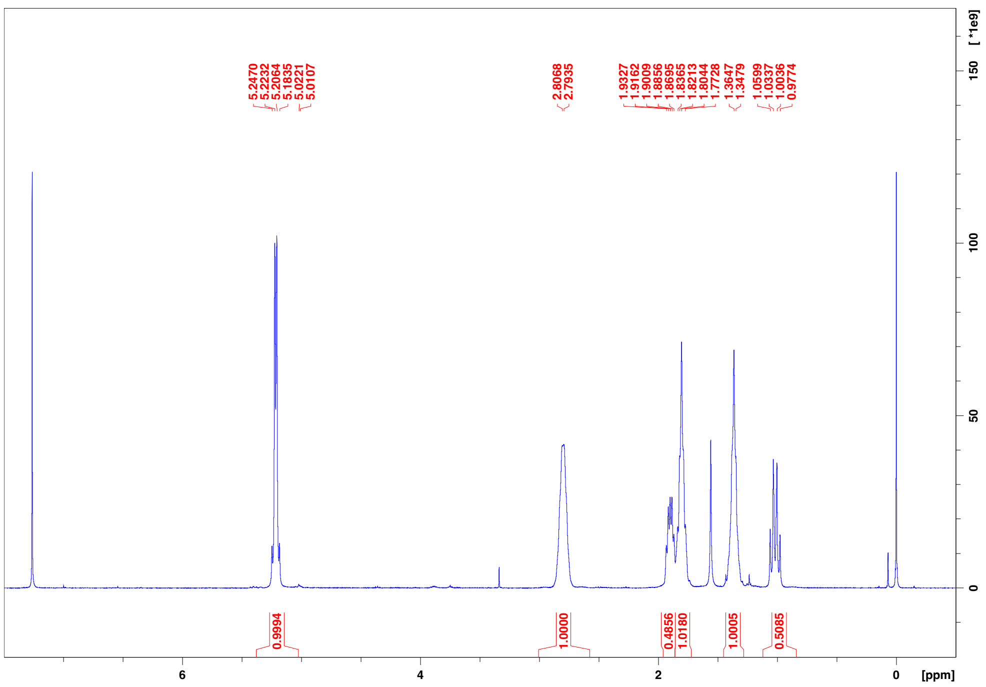

Figure S7. ^{1}H NMR (400 MHz, $CDCl_3$) spectrum of **Table 1** entry **6b** with 99% *cis*-alkenes in polymer backbone.

5.3 Overlaid GPC Chromatograms over Six Hour Degradation

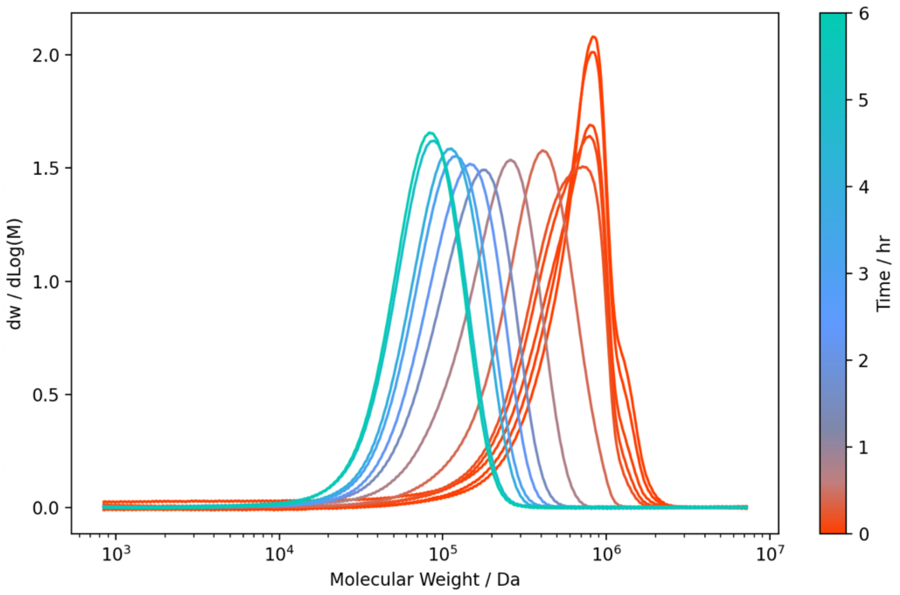

Figure S8. Overlaid molecular weight distribution of **Table 1** entry **1a** over six cumulative hours of ultrasonic degradation (compare to **Figure 3A** top).

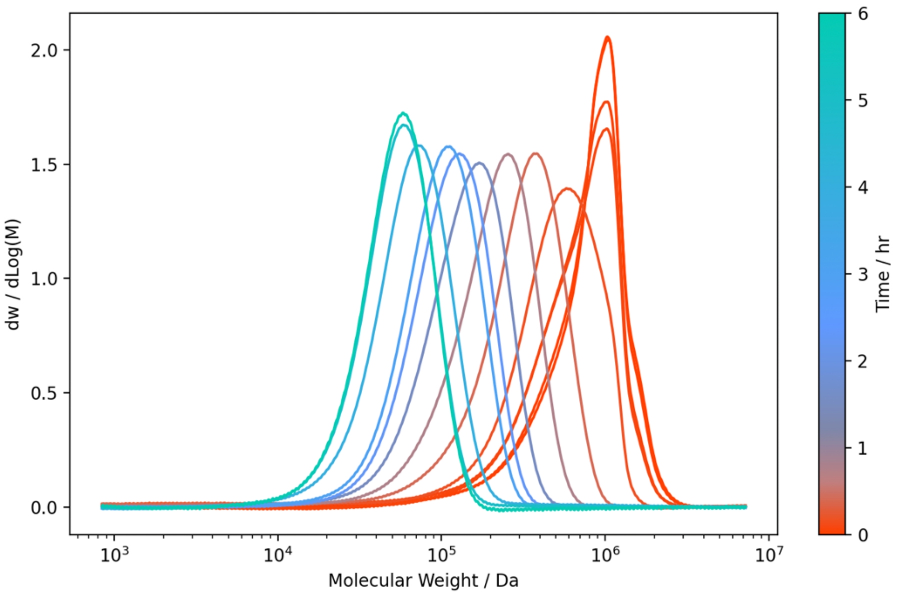

Figure S9. Overlaid molecular weight distribution of **Table 1** entry **1b** over six cumulative hours of ultrasonic degradation (compare to **Figure 3A** top).

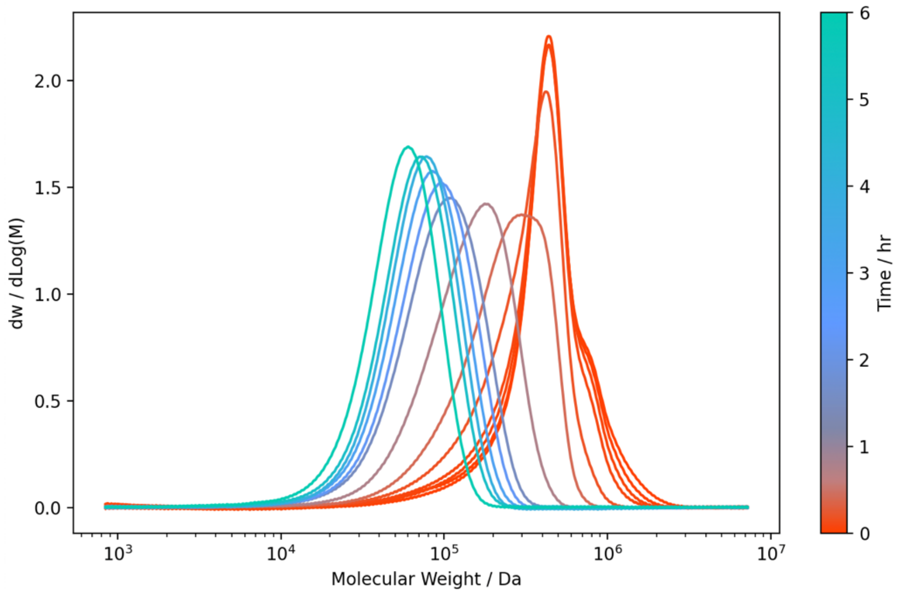

Figure S10. Overlaid molecular weight distribution of **Table 1** entry **3a** over six cumulative hours of ultrasonic degradation (compare to **Figure 3A** middle).

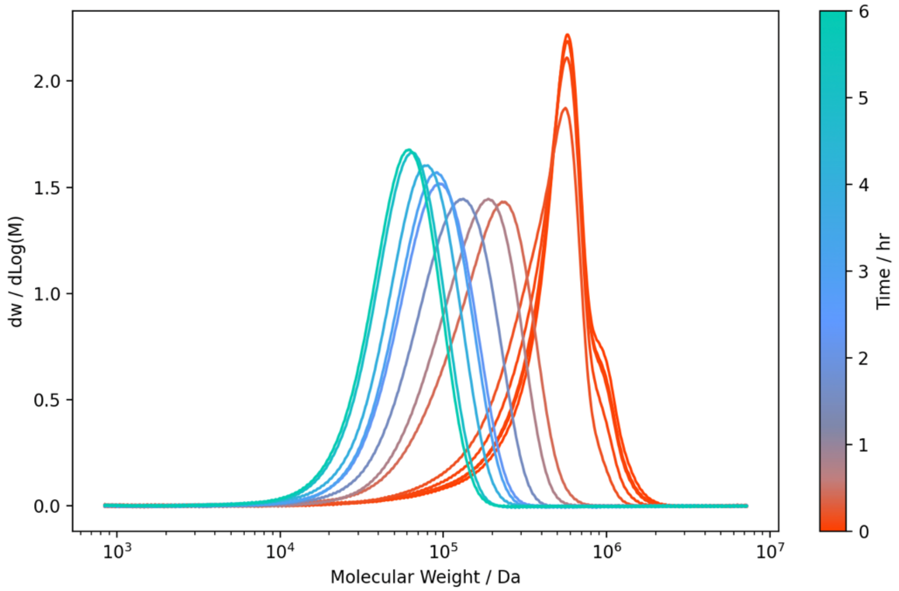

Figure S11. Overlaid molecular weight distribution of **Table 1** entry **3b** over six cumulative hours of ultrasonic degradation (compare to **Figure 3A** middle).

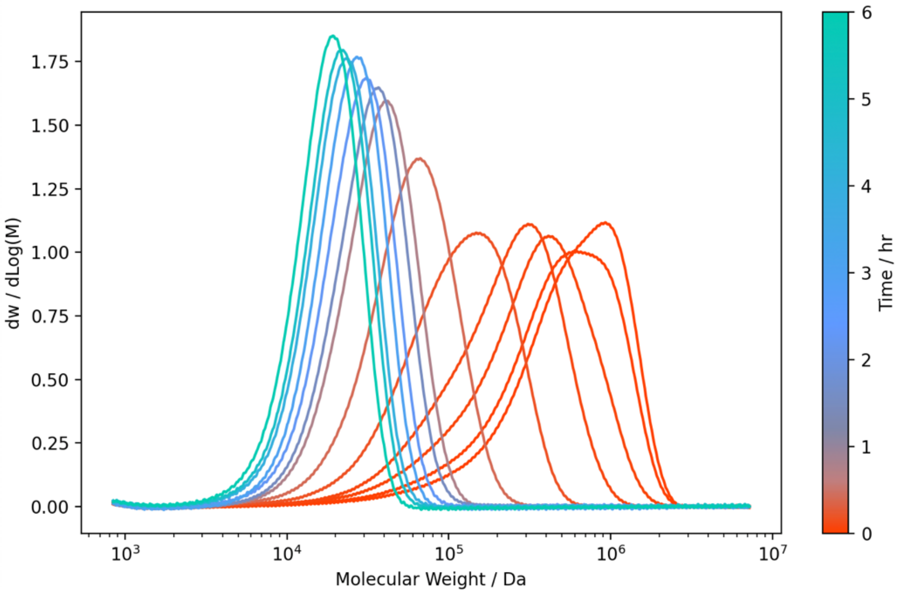

Figure S12. Overlaid molecular weight distribution of **Table 1** entry **5b** over six cumulative hours of ultrasonic degradation (compare to **Figure 3A** bottom).

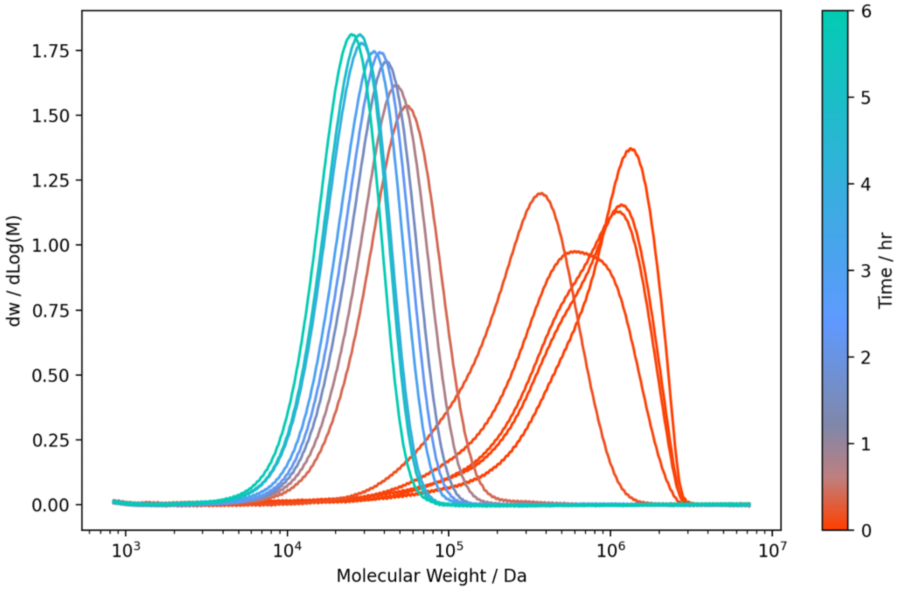

Figure S13. Overlaid molecular weight distribution of **Table 1** entry **5c** over six cumulative hours of ultrasonic degradation (compare to **Figure 3A** bottom).

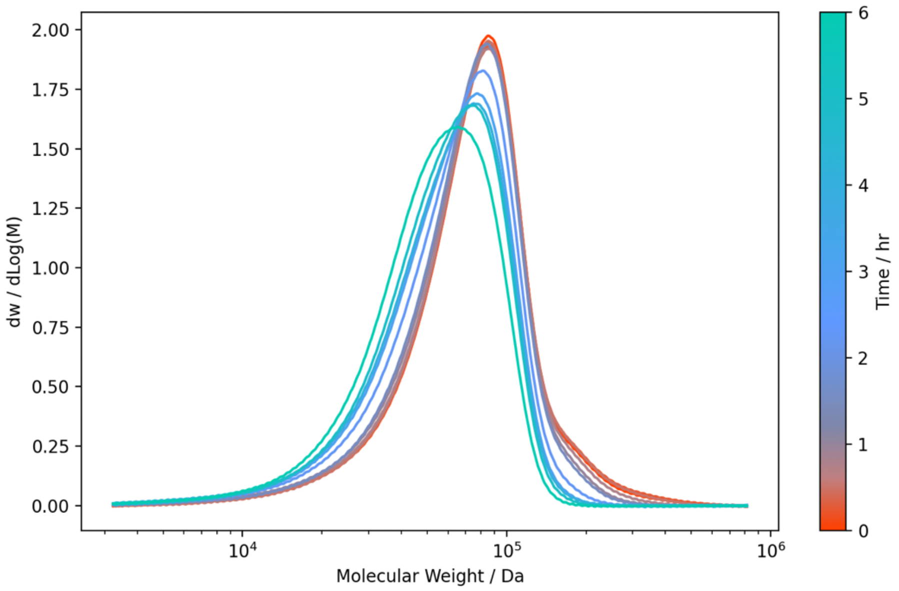

Figure S14. Overlaid molecular weight distribution of **Table 1** entry **2a** over six cumulative hours of ultrasonic degradation (compare to **Figure 3B** top).

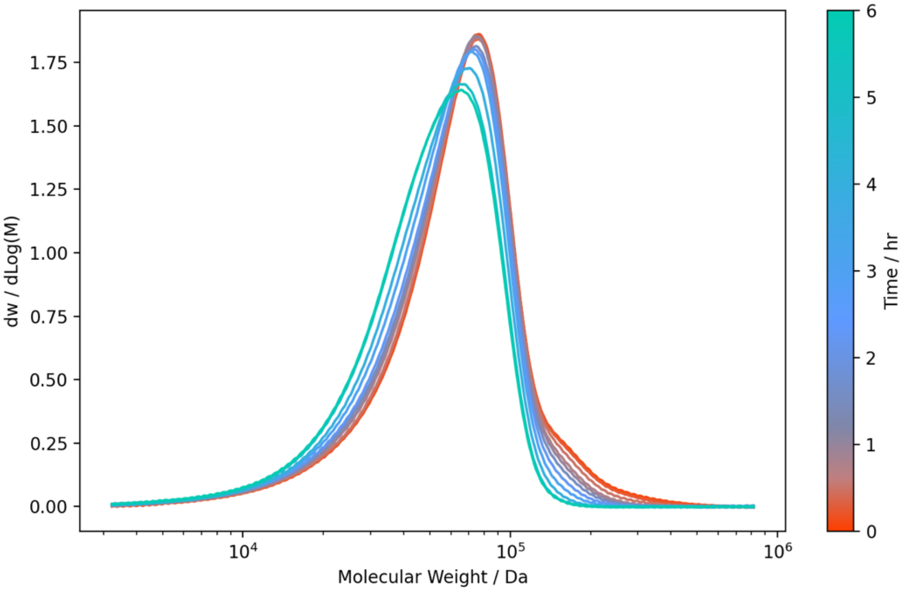

Figure S15. Overlaid molecular weight distribution of **Table 1** entry **2c** over six cumulative hours of ultrasonic degradation (compare to **Figure 3B** top).

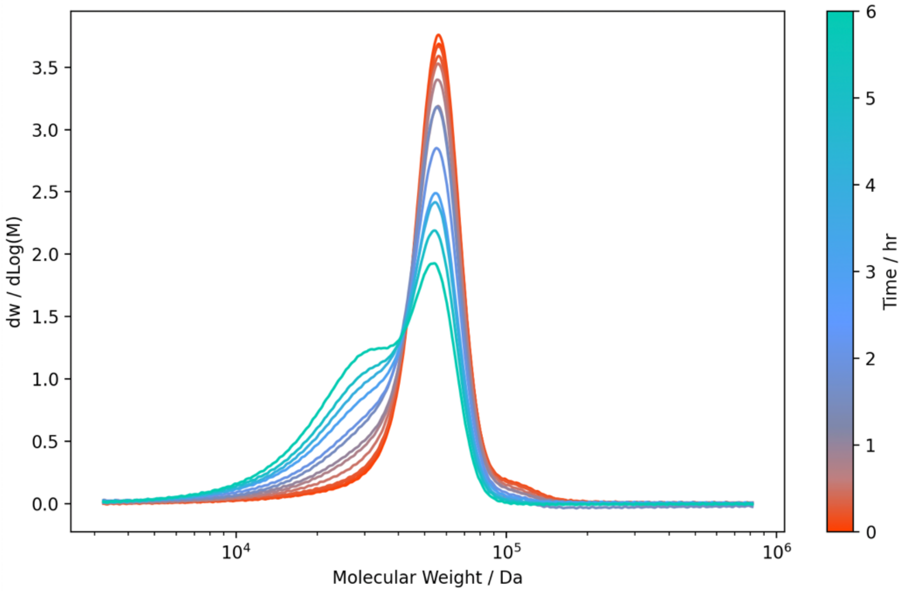

Figure S16. Overlaid molecular weight distribution of **Table 1** entry **4a** over six cumulative hours of ultrasonic degradation (compare to **Figure 3B** middle).

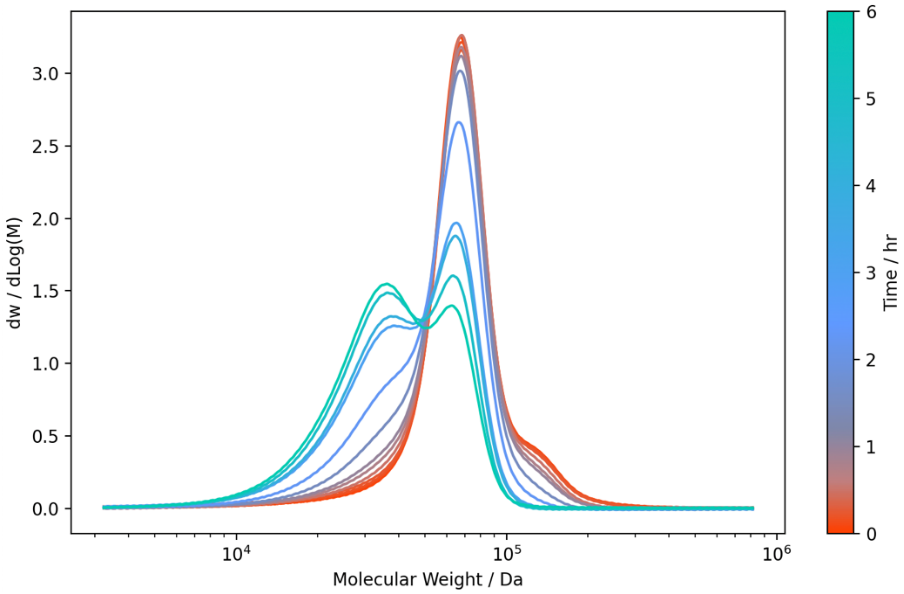

Figure S17. Overlaid molecular weight distribution of **Table 1** entry **4b** over six cumulative hours of ultrasonic degradation (compare to **Figure 3B** middle).

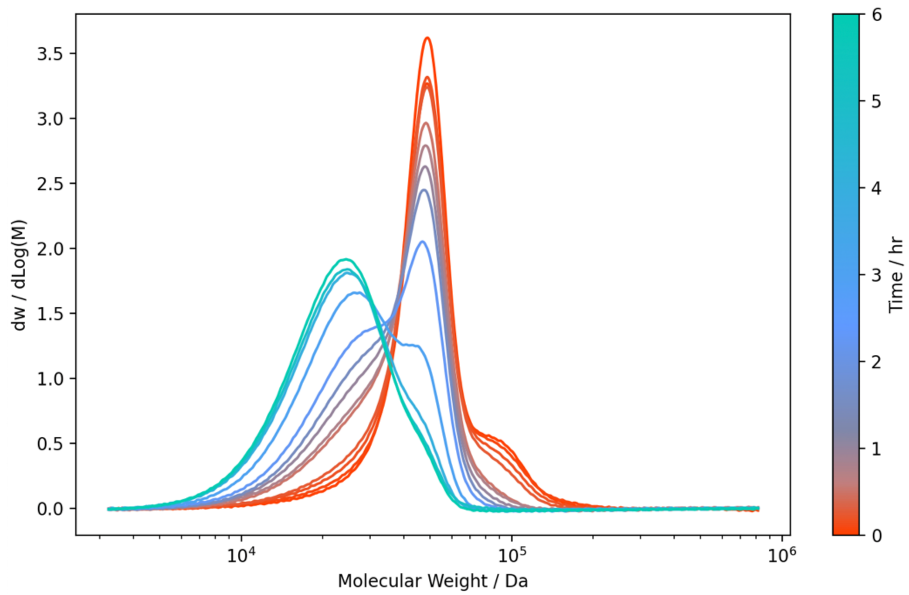

Figure S18. Overlaid molecular weight distribution of **Table 1** entry **6a** over six cumulative hours of ultrasonic degradation (compare to **Figure 3B** bottom).

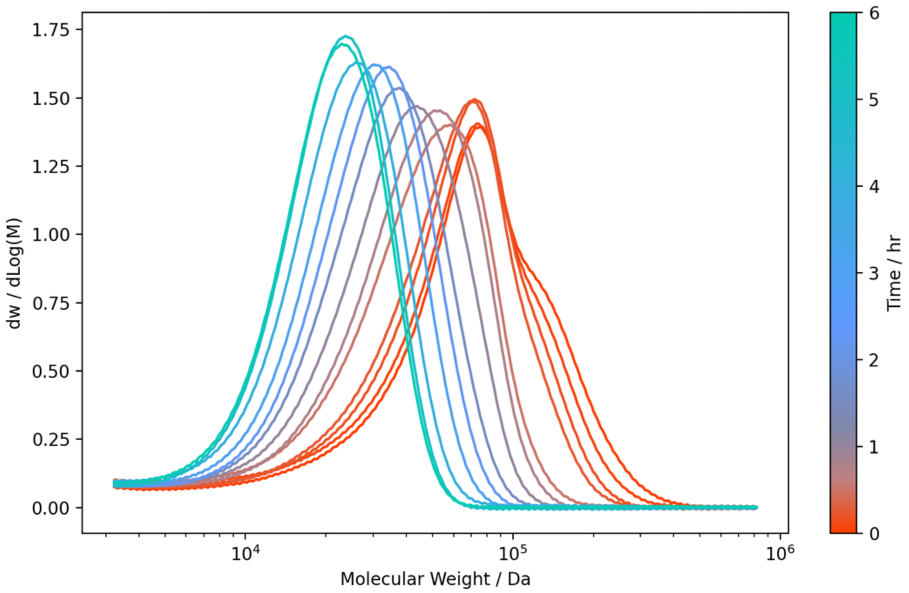

Figure S19. Overlaid molecular weight distribution of **Table 1** entry **6c** over six cumulative hours of ultrasonic degradation (compare to **Figure 3B** bottom).

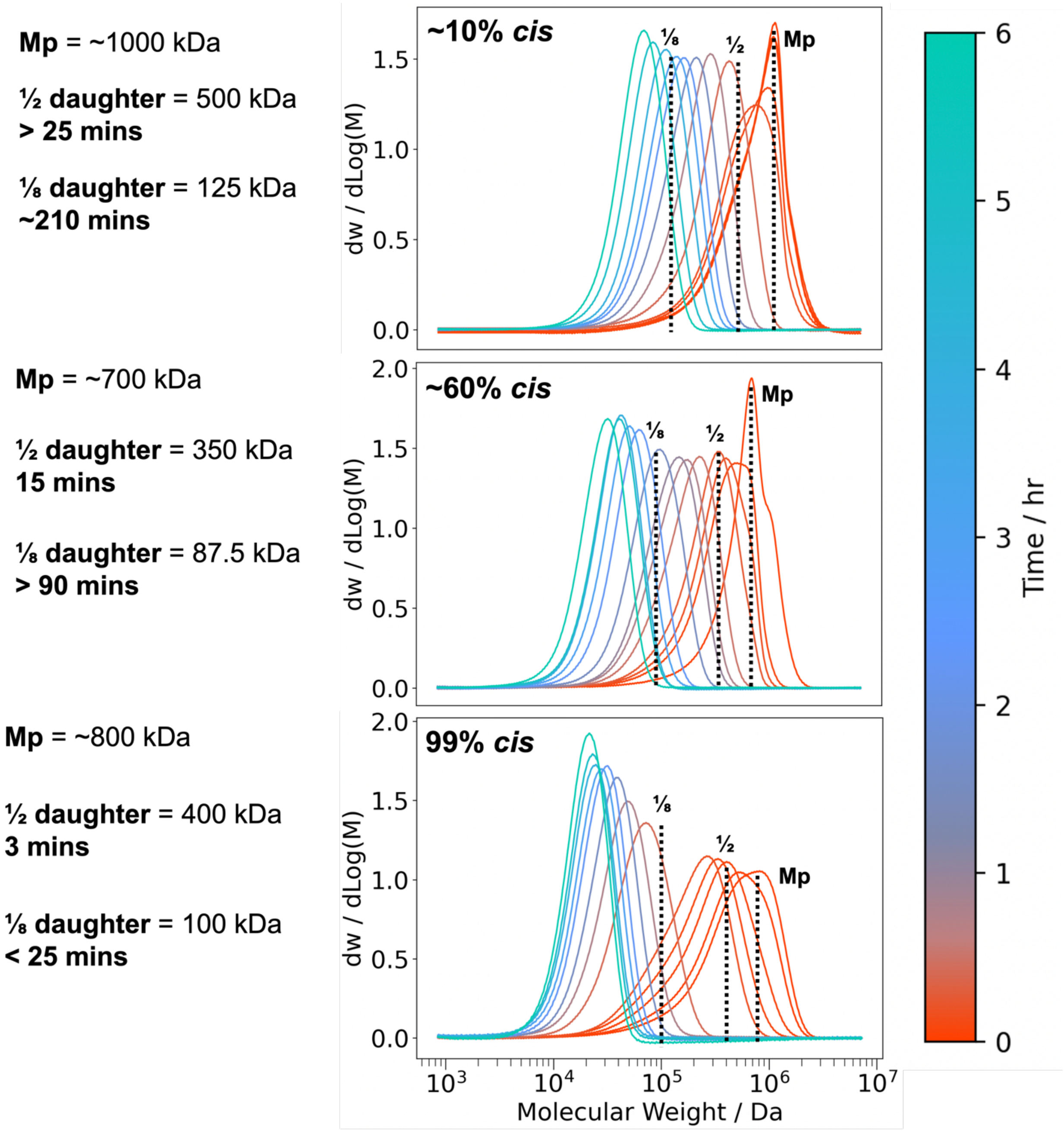

Figure S20. Overlaid molecular weight distributions of **Table 1** entries **1c**, **3c**, **5a** (top to bottom) with DP = 5000 (Mw ~700 kDa) with annotations for ½ and ⅛ daughter fragments and sonication time required to reach each fragment.

5.4 Scission Cycle Analysis

Scission cycle was calculated as previously reported[2] by the following formula:

$$(\ln(Mn_0) - \ln(Mn_t)) / \ln(2)$$

Where Mn_0 is the initial number-averaged molecular weight of the polymer and Mn_t is the number-averaged molecular weight of the polymer at sonication time t.

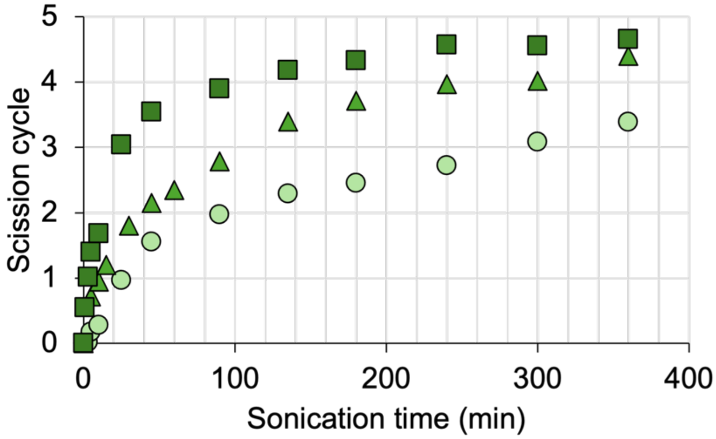

Figure S21. Polymer scission cycle as a function of sonication time for polymers **1c** (light-green circle), **3c** (medium-green triangle), **5a** (dark-green squares) (same polymers as presented in main text **Figures 3A and 4A**).

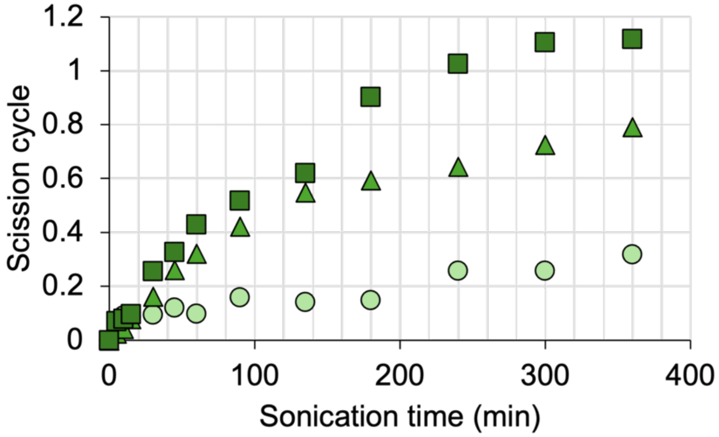

Figure S22. Polymer scission cycle as a function of sonication time for polymers **2b** (light-green circle), **4c** (medium-green triangle), **6b** (dark-green squares) (same polymers as presented in main text **Figures 3B and 4B**).

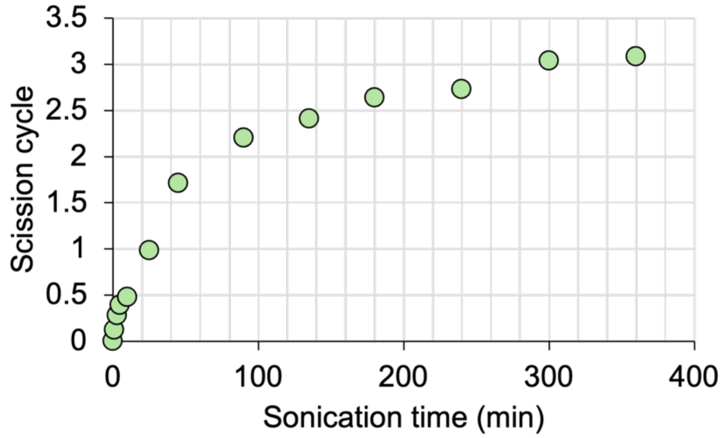

Figure S23. Polymer scission cycle as a function of sonication time for **Table 1** entry **1a**.

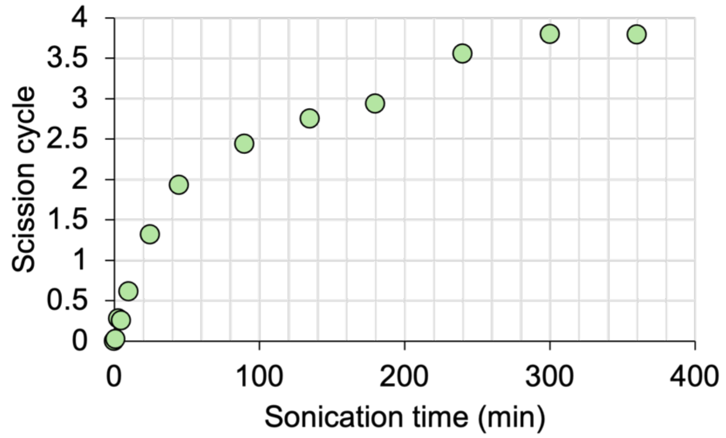

Figure S24. Polymer scission cycle as a function of sonication time for **Table 1** entry **1b**.

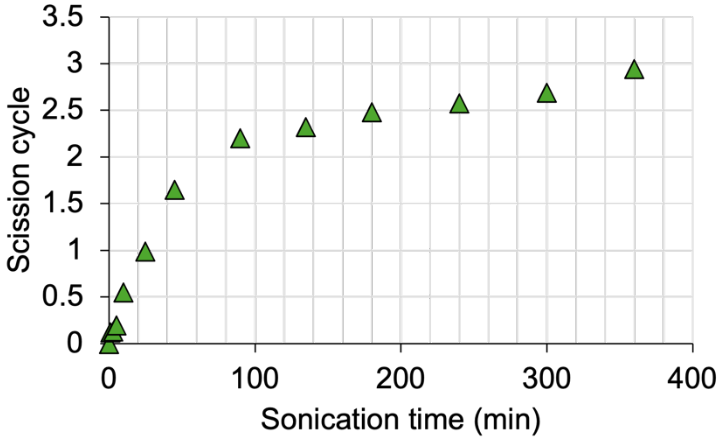

Figure S25. Polymer scission cycle as a function of sonication time for **Table 1** entry **3a**.

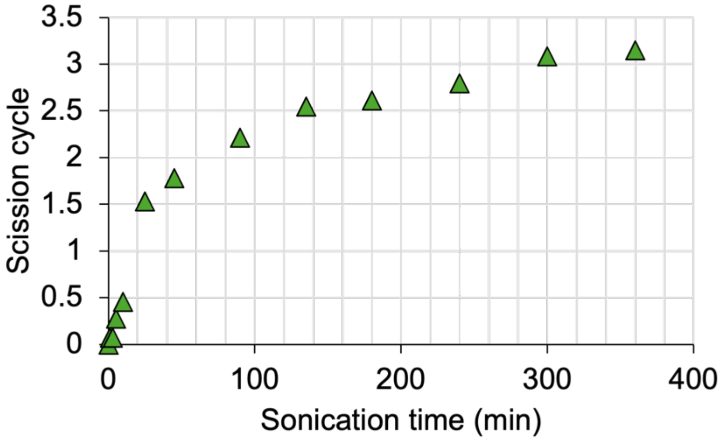

Figure S26. Polymer scission cycle as a function of sonication time for **Table 1** entry **3b**.

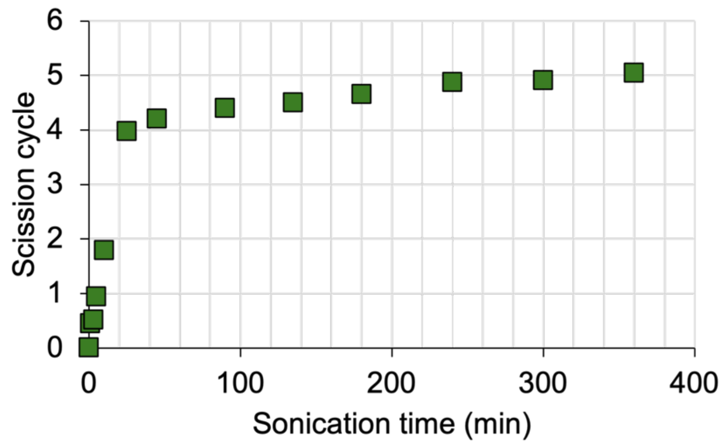

Figure S27. Polymer scission cycle as a function of sonication time for **Table 1** entry **5b**.

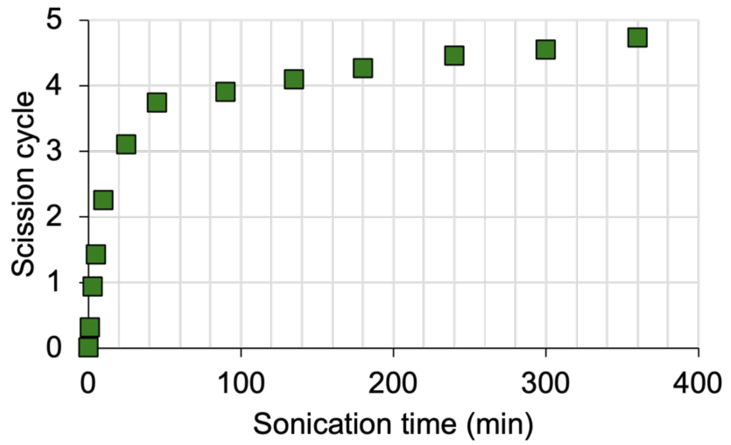

Figure S28. Polymer scission cycle as a function of sonication time for **Table 1** entry **5c**.

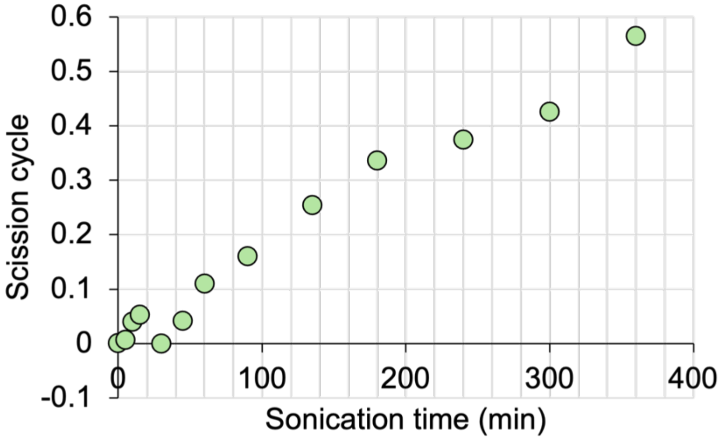

Figure S29. Polymer scission cycle as a function of sonication time for **Table 1** entry **2a**.

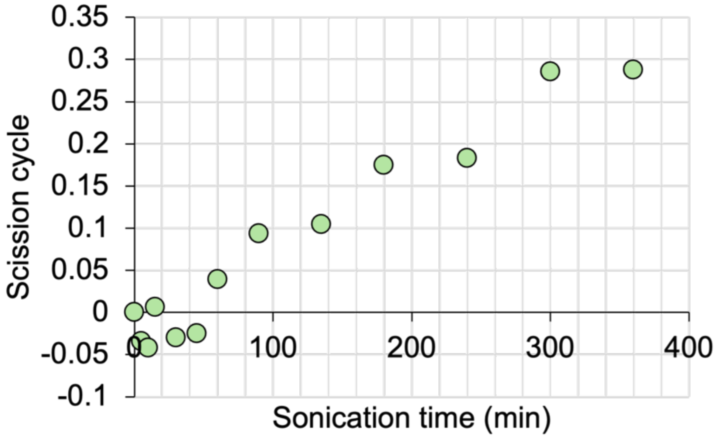

Figure S30. Polymer scission cycle as a function of sonication time for **Table 1** entry **2c**.

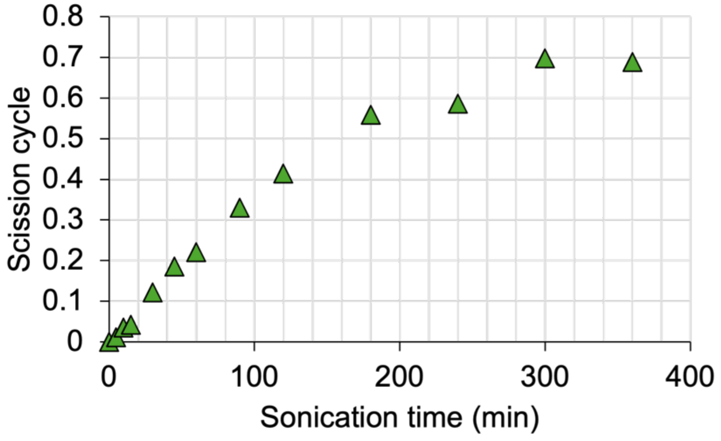

Figure S31. Polymer scission cycle as a function of sonication time for **Table 1** entry **4a**.

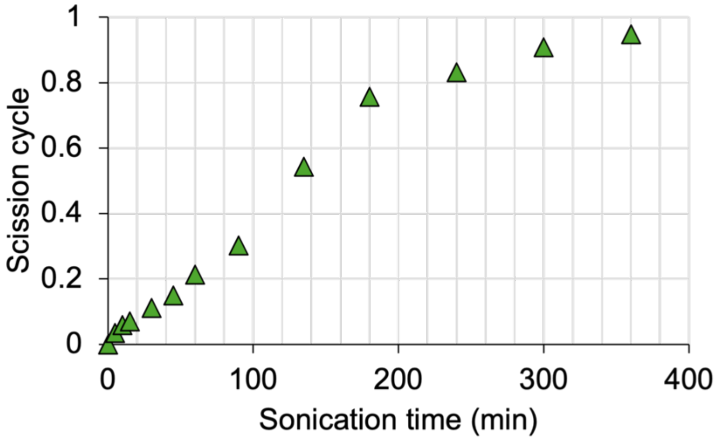

Figure S32. Polymer scission cycle as a function of sonication time for **Table 1** entry **4b**.

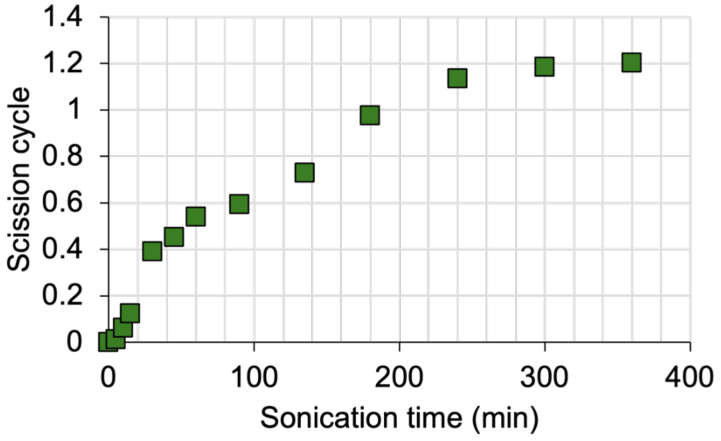

Figure S33. Polymer scission cycle as a function of sonication time for **Table 1** entry **6a**.

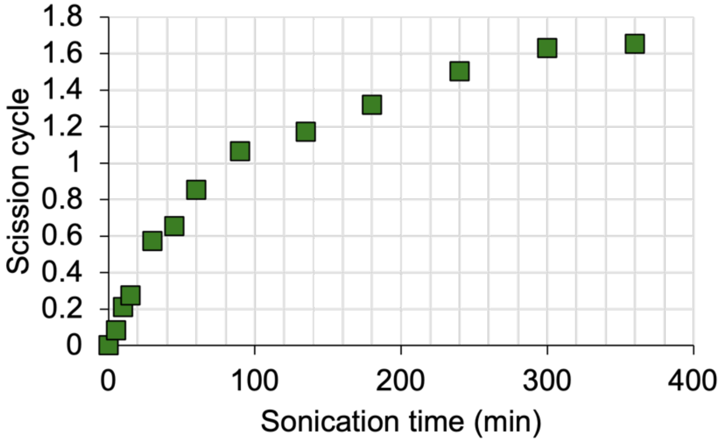

Figure S34. Polymer scission cycle as a function of sonication time for **Table 1** entry **6c**.

5.5 Ultrasonication Degradation Controls

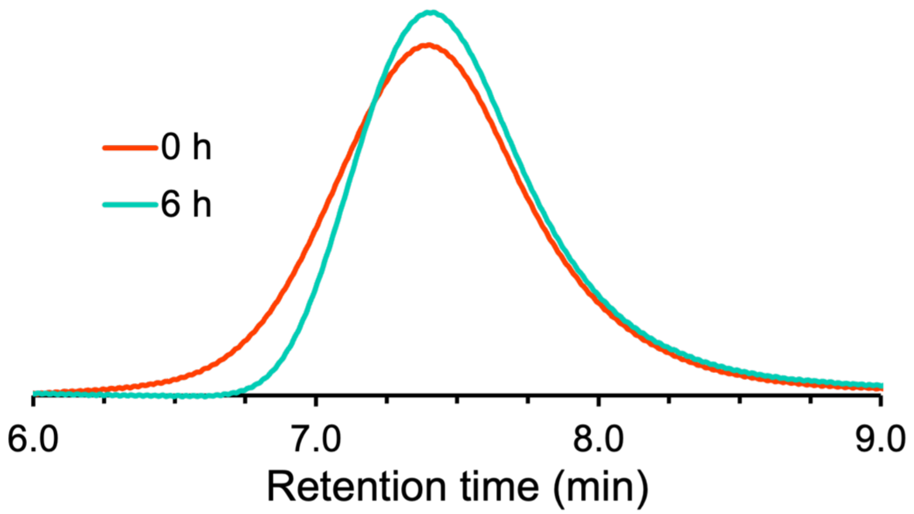

Figure S35. Overlaid GPC molecular weight distribution as a function of retention time for a ~25 kDa M_w polymer with ~10% *cis*-alkenes (Polymer **7** from **Table S9**) before and after six hours of continuous ultrasonication with peak areas normalized.

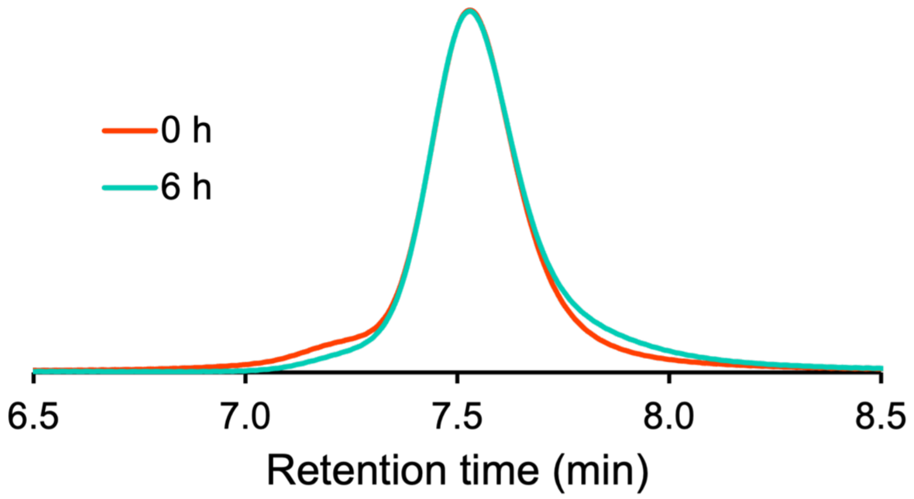

Figure S36. Overlaid GPC molecular weight distribution as a function of retention time for a ~25 kDa M_w polymer with ~60% *cis*-alkenes (Polymer **8** from **Table S9**) before and after six hours of continuous ultrasonication with peak areas normalized.

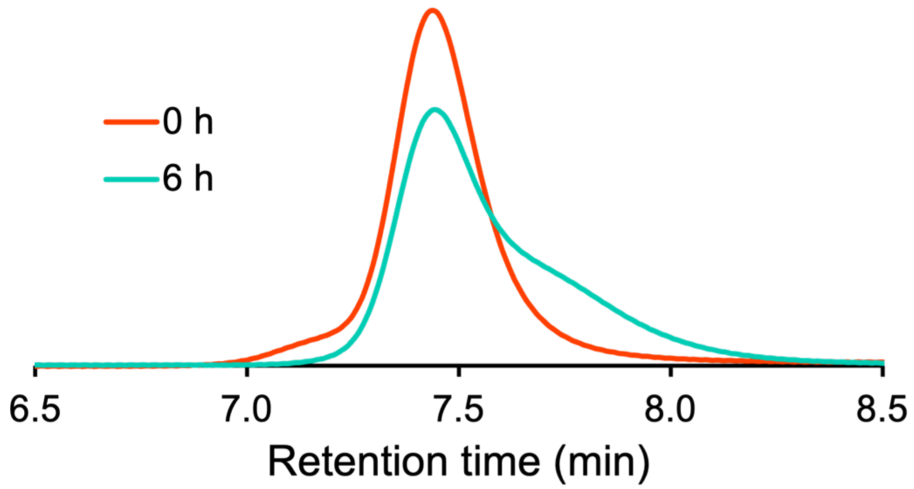

Figure S37. Overlaid GPC molecular weight distribution as a function of retention time for a ~25 kDa M_w polymer with ~100% *cis*-alkenes (Polymer **9** from **Table S9**) before and after six hours of continuous ultrasonication with peak areas normalized.

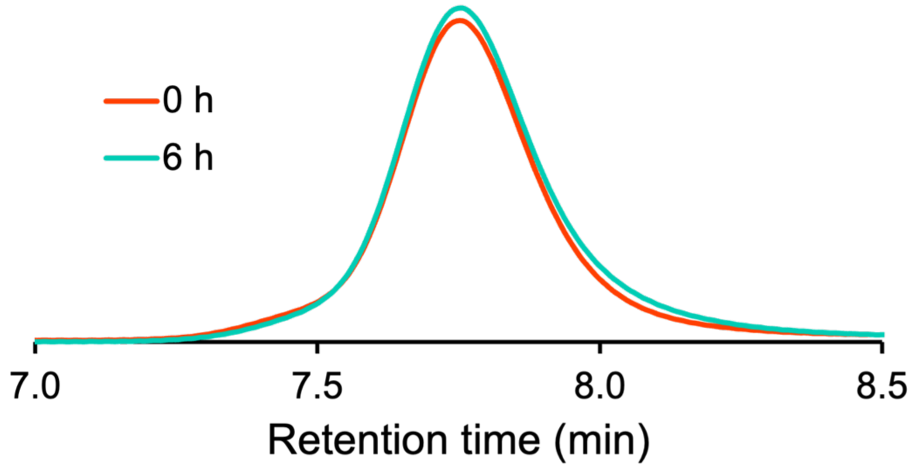

Figure S38. Overlaid GPC molecular weight distribution as a function of retention time for a ~12.5 kDa M_w polymer with ~100% *cis*-alkenes (Polymer **10** from **Table S9**) before and after six hours of continuous ultrasonication with peak areas normalized.

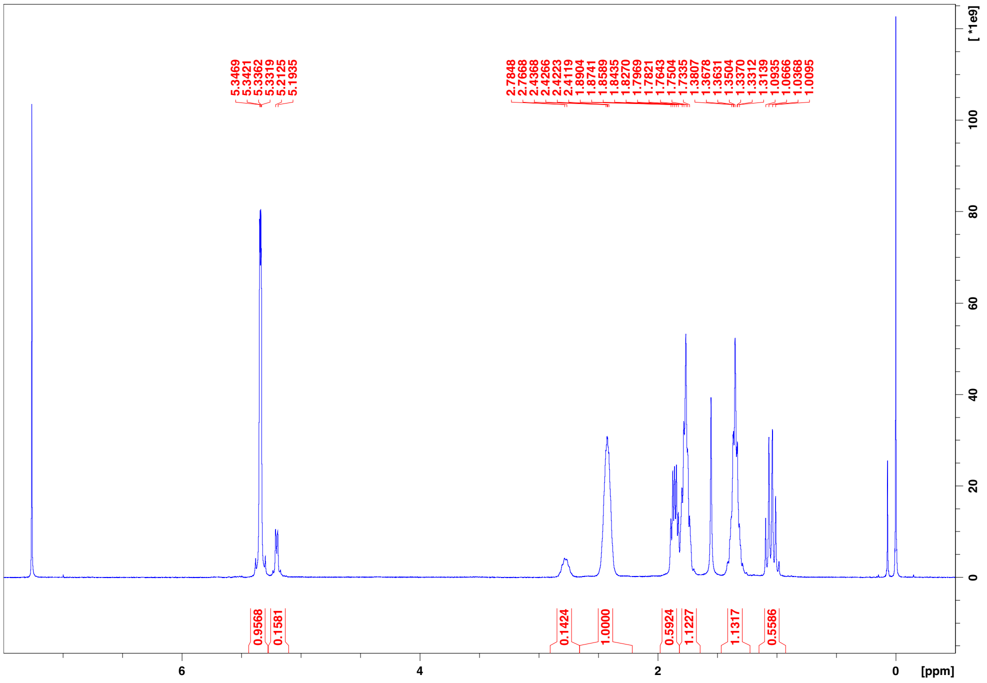

Figure S39. ^1H NMR (400 MHz, $CDCl_3$) spectrum of **Table S1** entry **3r** with ~10% *cis*-alkenes in polymer backbone after 24 hours of continuous ultrasonic degradation.

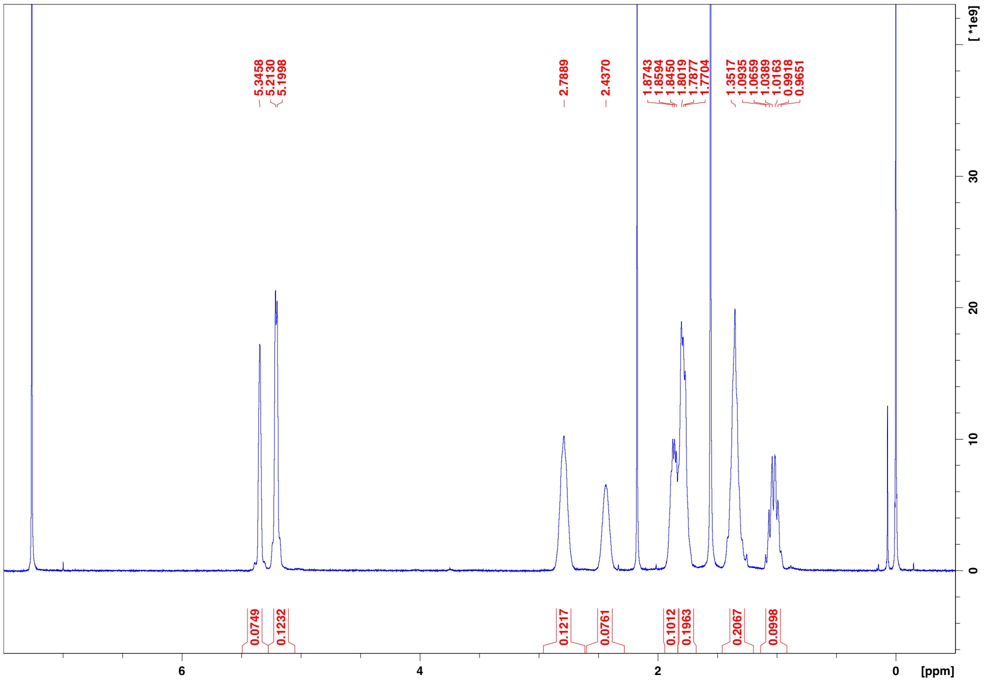

Figure S40. ^1H NMR (400 MHz, $CDCl_3$) spectrum of **Table 1** entry **3c** with ~60% *cis*-alkenes in polymer backbone after 24 hours of continuous ultrasonic degradation.

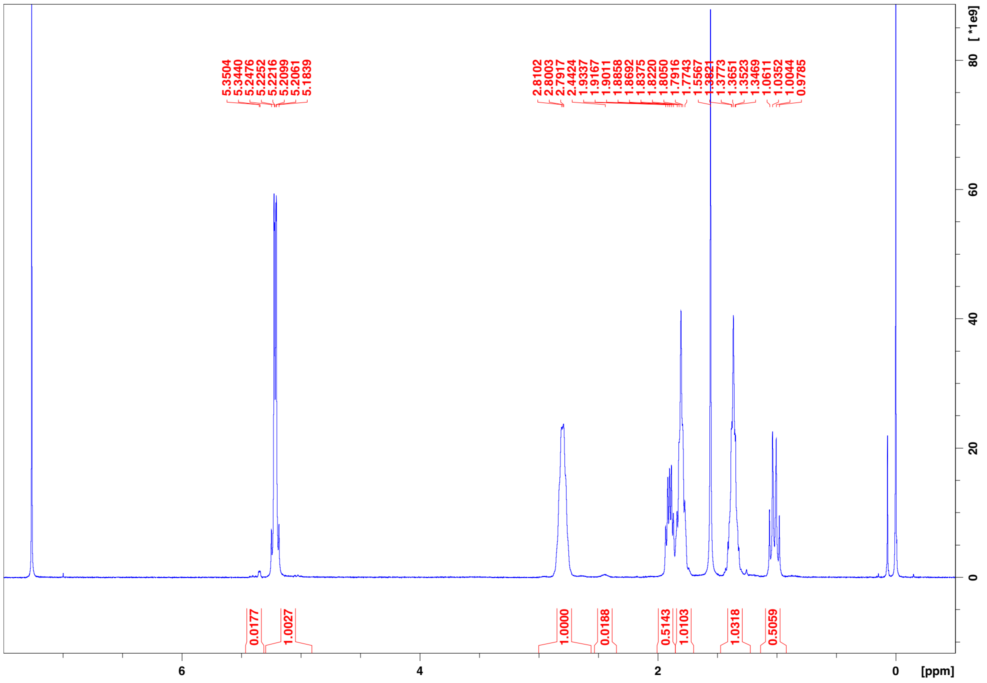

Figure S41. ^{1}H NMR (400 MHz, $CDCl_3$) spectrum of **Table 1** entry **5a** with 99% *cis*-alkenes in polymer backbone after 24 hours of continuous ultrasonic degradation.

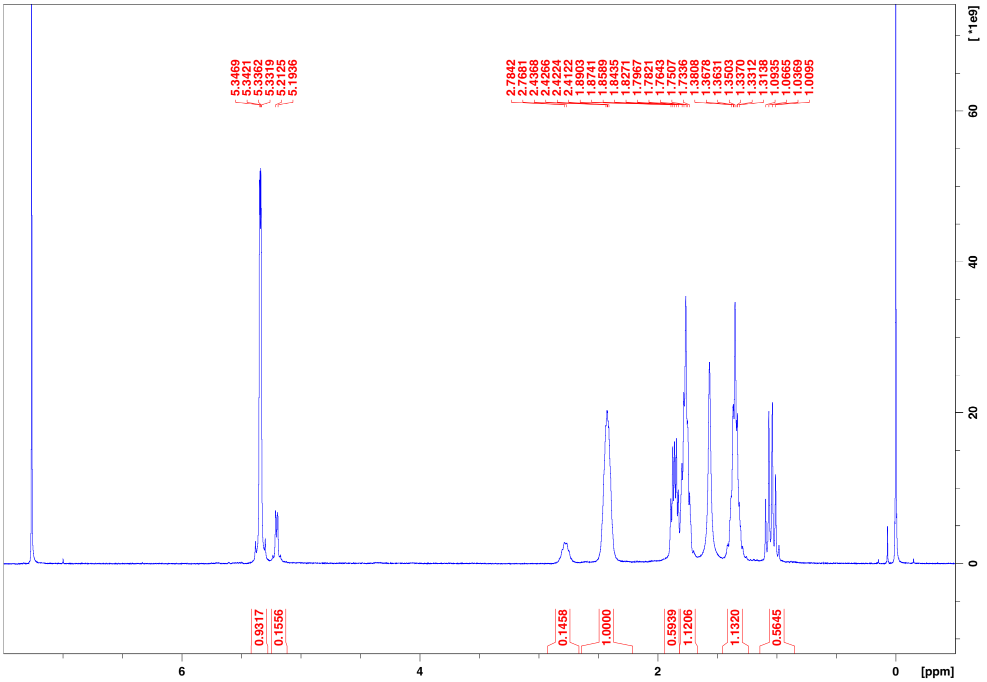

Figure S42. ^{1}H NMR (400 MHz, $CDCl_3$) spectrum of **Table 1** entry **2b** with ~12% *cis*-alkenes in polymer backbone after 24 hours of continuous ultrasonic degradation.

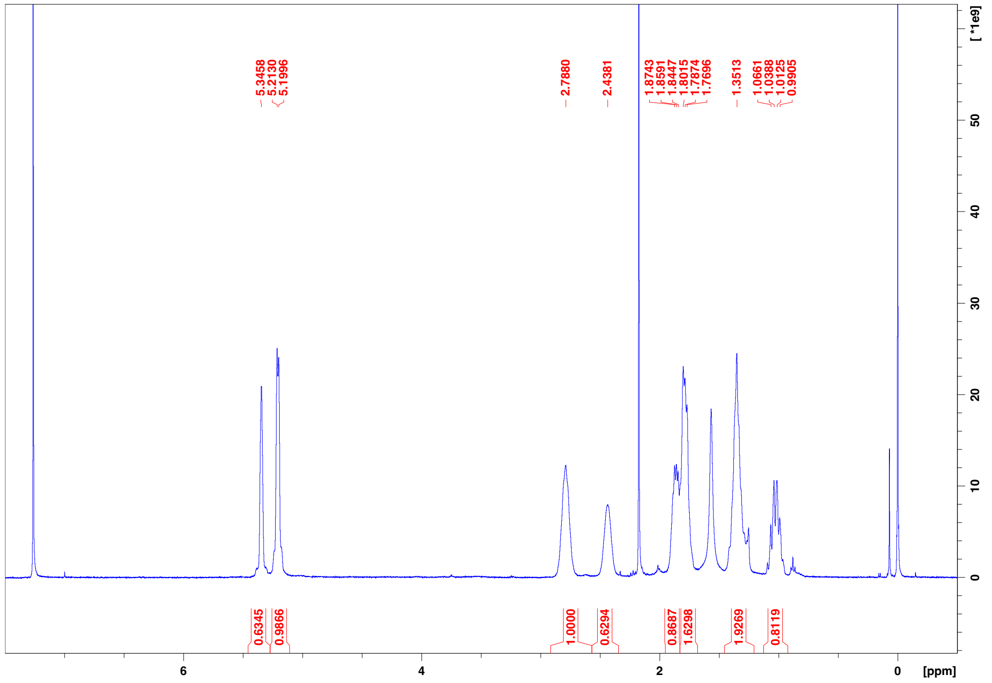

Figure S43. ^{1}H NMR (400 MHz, $CDCl_3$) spectrum of **Table 1** entry **4c** with ~60% *cis*-alkenes in polymer backbone after 24 hours of continuous ultrasonic degradation.

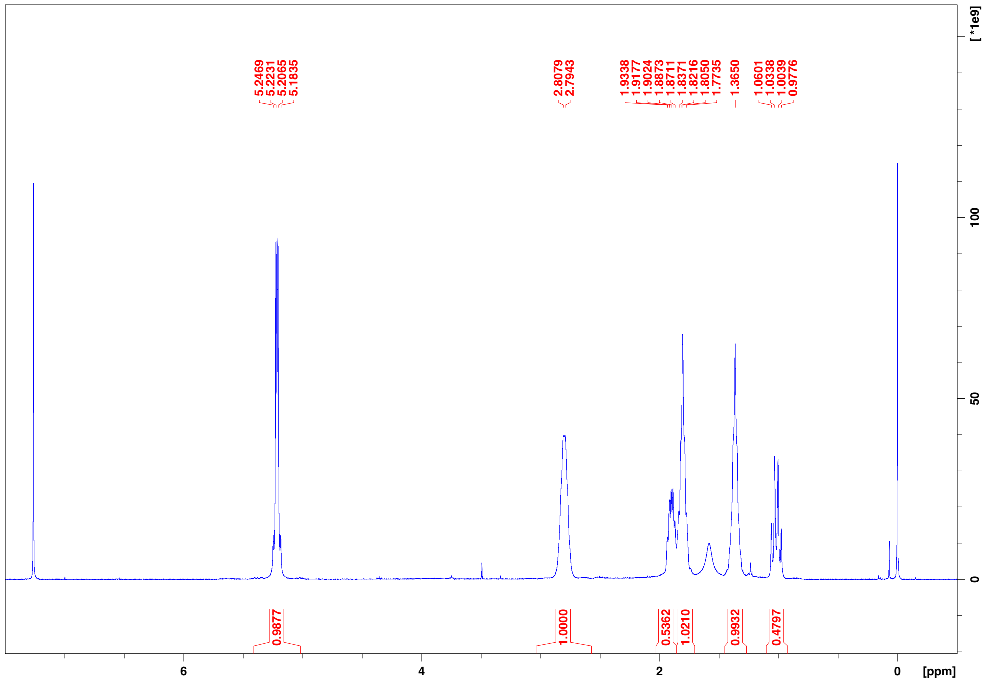

Figure S44. ^{1}H NMR (400 MHz, $CDCl_3$) spectrum of **Table 1** entry **6b** with 99% *cis*-alkenes in polymer backbone after 24 hours of continuous ultrasonic degradation.

5.6 Initial Peak Molecular Weight Intensity Analysis

We analyzed the change in initial M_p intensity over sonication time by measuring the refractive index (RI) signal at the GPC retention time corresponding to the M_p of the parent polymer across all degradation aliquots. The RI values were then normalized twice: first by peak area to account for concentration differences across samples, and then by the parent M_p intensity to ensure that the initial RI of the M_p was equal to 1.0 at $t = 0$ min for all samples.

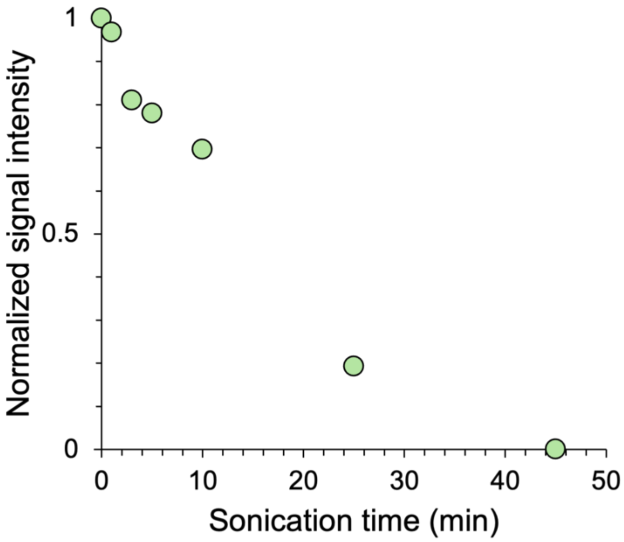

Figure S45. Normalized refractive index signal intensity of the initial peak molecular weight, M_p, in the molecular weight distribution of **Table 1** entry **1a** over six cumulative hours of ultrasonic degradation (compare to **Figure 4A**).

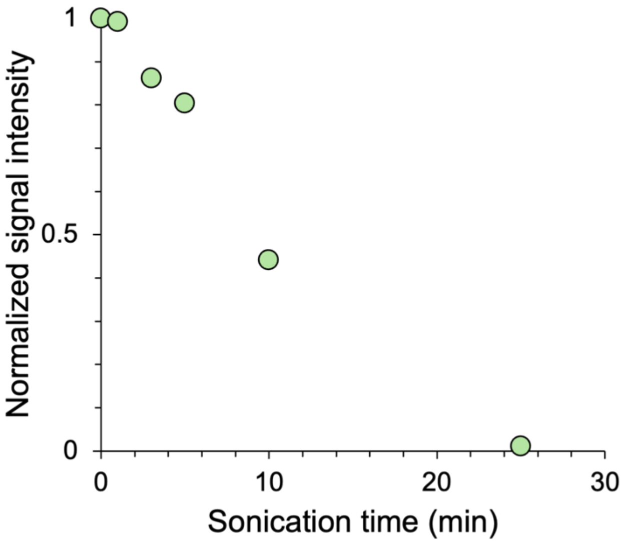

Figure S46. Normalized refractive index signal intensity of the initial peak molecular weight, M_p, in the molecular weight distribution of **Table 1** entry **1b** over six cumulative hours of ultrasonic degradation (compare to **Figure 4A**).

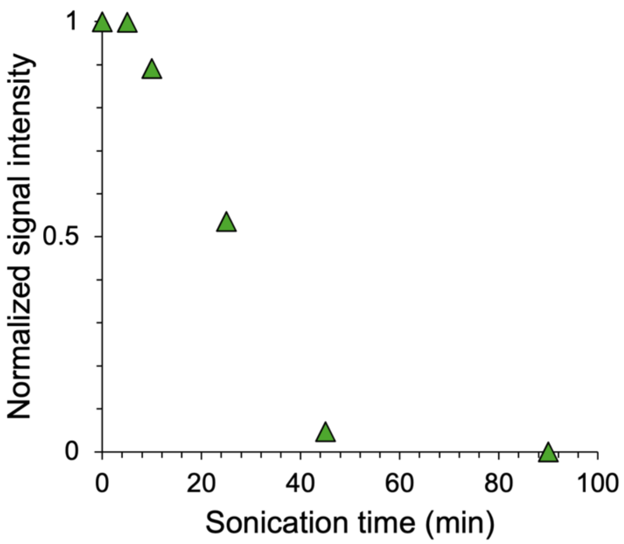

Figure S47. Normalized refractive index signal intensity of the initial peak molecular weight, M_p, in the molecular weight distribution of **Table 1** entry **3a** over six cumulative hours of ultrasonic degradation (compare to **Figure 4A**).

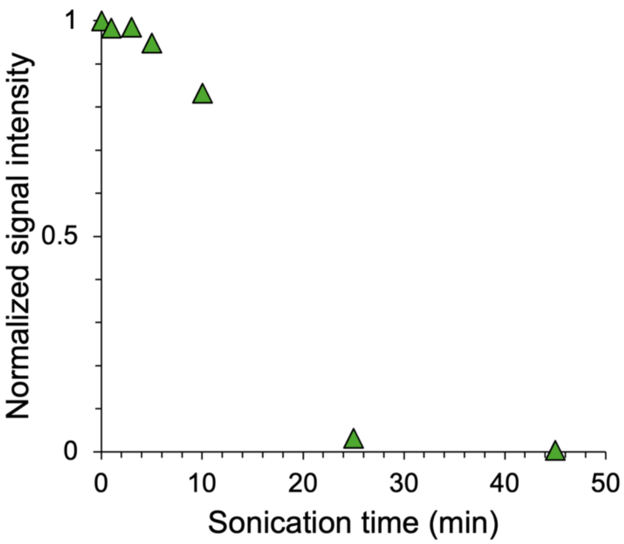

Figure S48. Normalized refractive index signal intensity of the initial peak molecular weight, M_p, in the molecular weight distribution of **Table 1** entry **3b** over six cumulative hours of ultrasonic degradation (compare to **Figure 4A**).

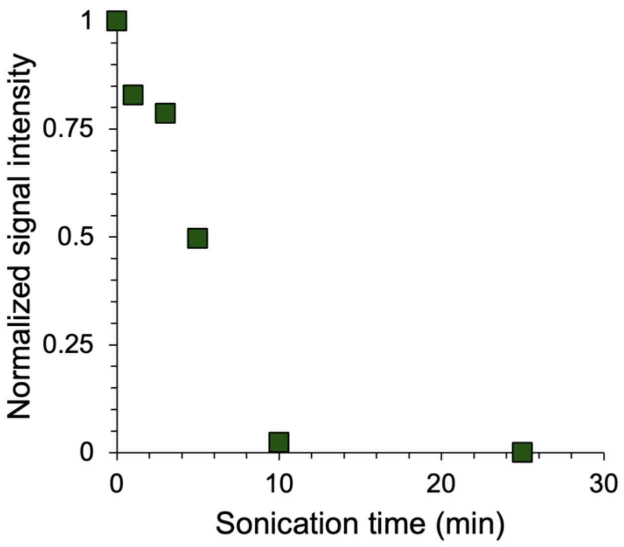

Figure S49. Normalized refractive index signal intensity of the initial peak molecular weight, M_p, in the molecular weight distribution of **Table 1** entry **5b** over six cumulative hours of ultrasonic degradation (compare to **Figure 4A**).

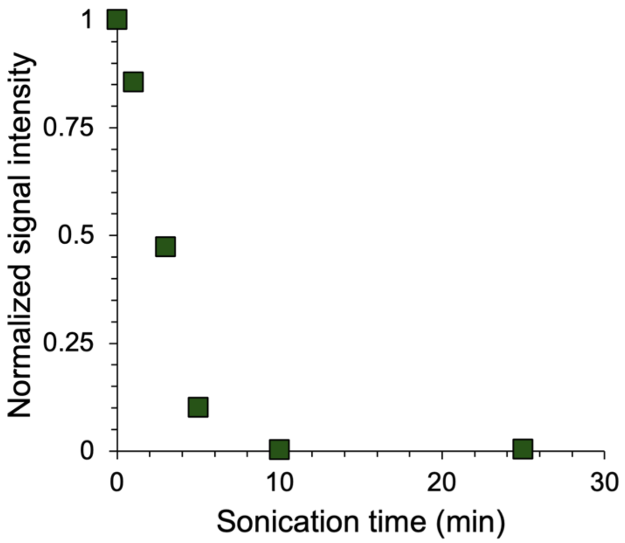

Figure S50. Normalized refractive index signal intensity of the initial peak molecular weight, M_p, in the molecular weight distribution of **Table 1** entry **5c** over six cumulative hours of ultrasonic degradation (compare to **Figure 4A**).

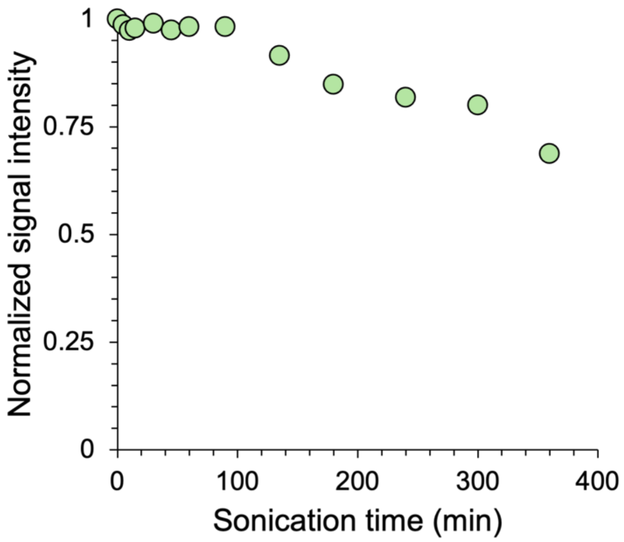

Figure S51. Normalized refractive index signal intensity of the initial peak molecular weight, M_p, in the molecular weight distribution of **Table 1** entry **2a** over six cumulative hours of ultrasonic degradation (compare to **Figure 4B**).

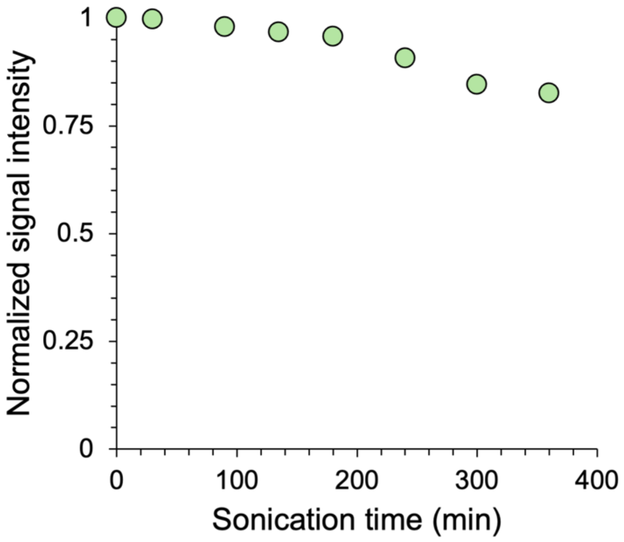

Figure S52. Normalized refractive index signal intensity of the initial peak molecular weight, M_p, in the molecular weight distribution of **Table 1** entry **2c** over six cumulative hours of ultrasonic degradation (compare to **Figure 4B**).

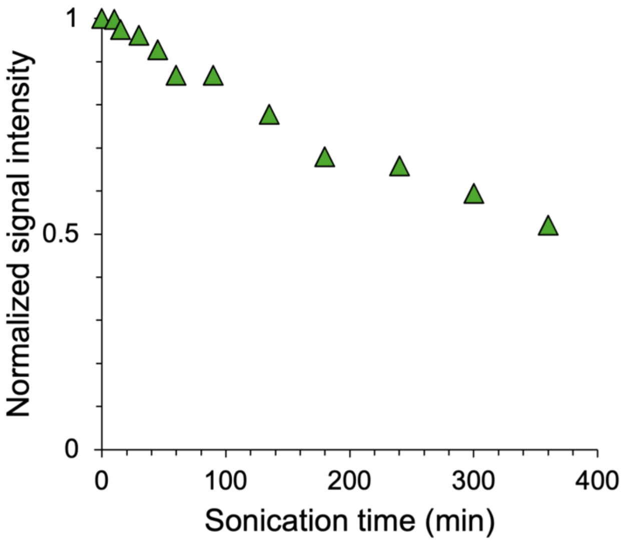

Figure S53. Normalized refractive index signal intensity of the initial peak molecular weight, M_p, in the molecular weight distribution of **Table 1** entry **4a** over six cumulative hours of ultrasonic degradation (compare to **Figure 4B**).

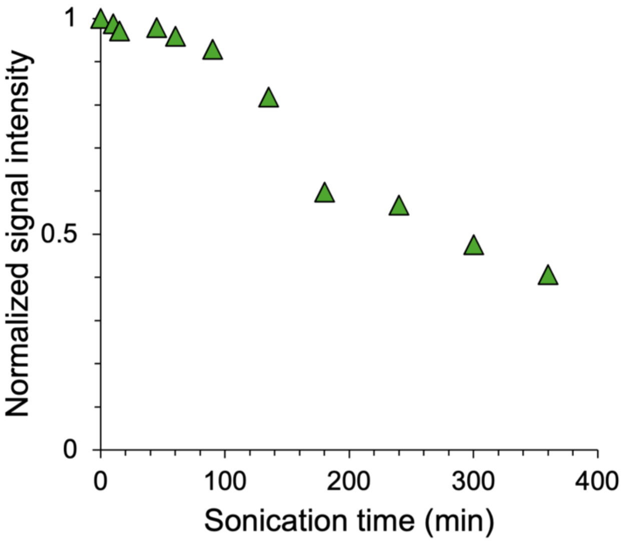

Figure S54. Normalized refractive index signal intensity of the initial peak molecular weight, M_p, in the molecular weight distribution of **Table 1** entry **4b** over six cumulative hours of ultrasonic degradation (compare to **Figure 4B**).

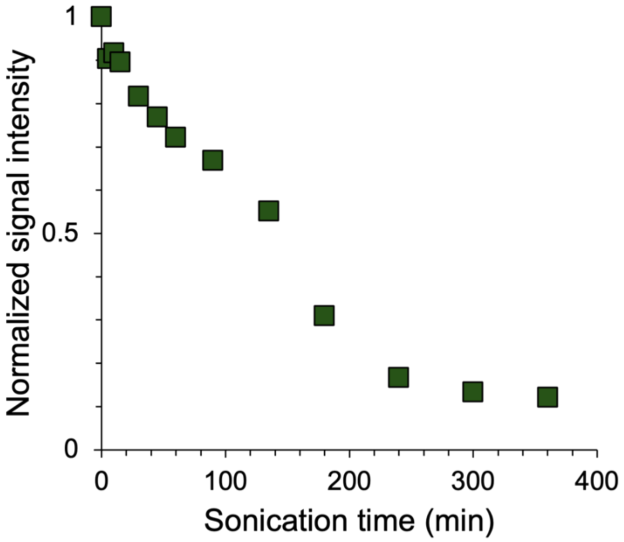

Figure S55. Normalized refractive index signal intensity of the initial peak molecular weight, M_p, in the molecular weight distribution of **Table 1** entry **6a** over six cumulative hours of ultrasonic degradation (compare to **Figure 4B**).

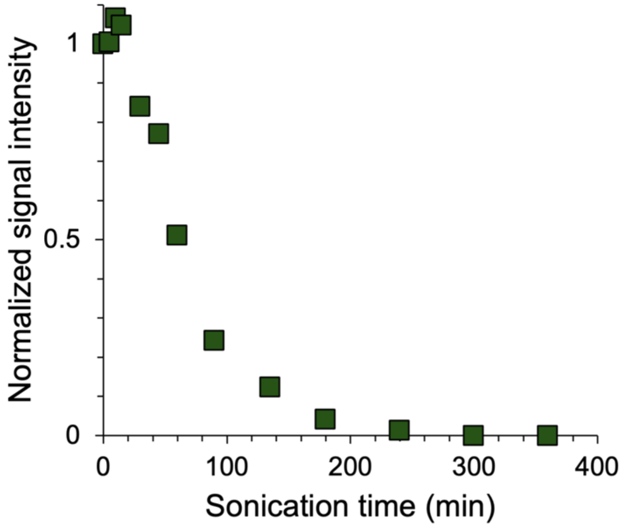

Figure S56. Normalized refractive index signal intensity of the initial peak molecular weight, M_p, in the molecular weight distribution of **Table 1** entry **6c** over six cumulative hours of ultrasonic degradation (compare to **Figure 4B**).

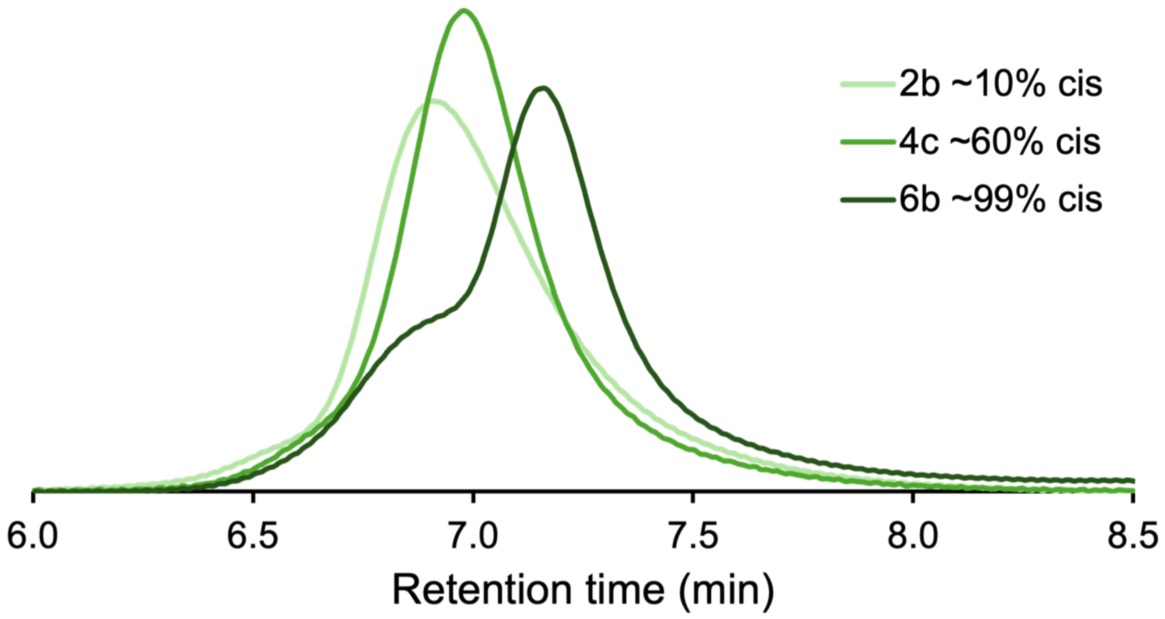

Figure S57. Overlaid molecular weight distributions normalized by peak area of **Table 1** entries **2b** (light green), **4c** (medium green), and **6b** (dark green) prior to ultrasonic degradation, showing the high molecular weight shoulder of **6b.**

5.7 Degradation Rate Analysis

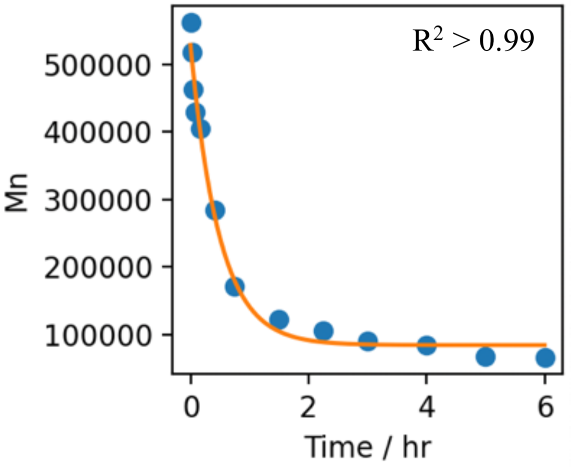

Figure S58. M_n and M_w values of **Table 1** entry **1a** over six hours of ultrasonic degradation fitted to exponential decay curves, corresponding to a degradation rate of 0.8722 min^{-1} (M_n) and 0.8497 min^{-1} (M_w).

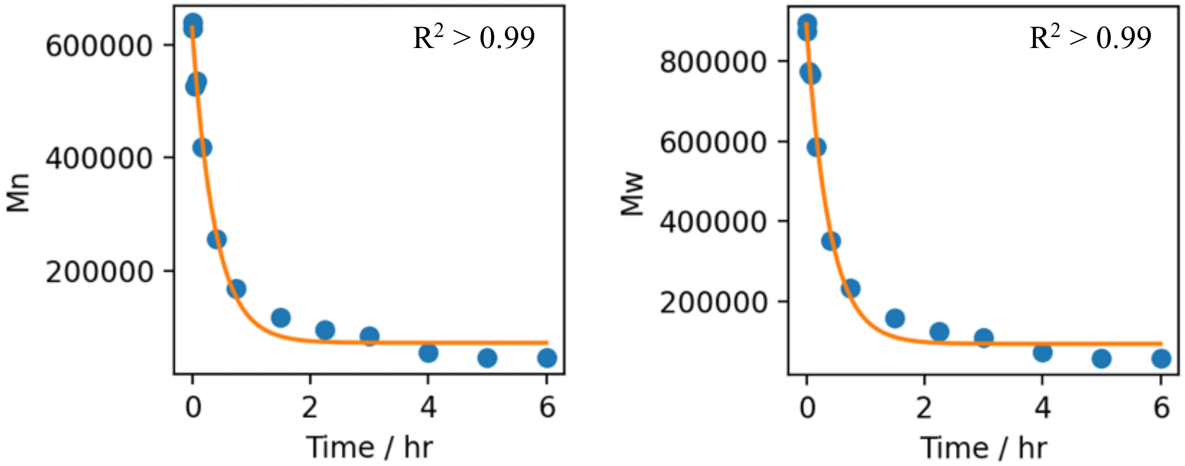

Figure S59 M_n and M_w values of **Table 1** entry **1b** over six hours of ultrasonic degradation fitted to exponential decay curves, corresponding to a degradation rate of 0.9245 min^{-1} (M_n) and 0.9232 min^{-1} (M_w).

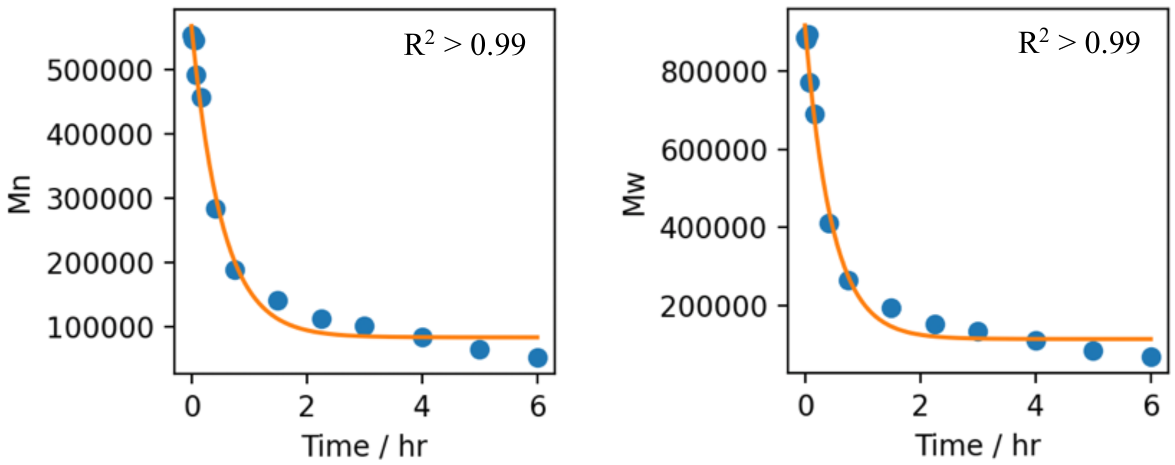

Figure S60. M_n and M_w values of **Table 1** entry **1c** over six hours of ultrasonic degradation fitted to exponential decay curves, corresponding to a degradation rate of 0.8481 min^{-1} (M_n) and 0.8830 min^{-1} (M_w).

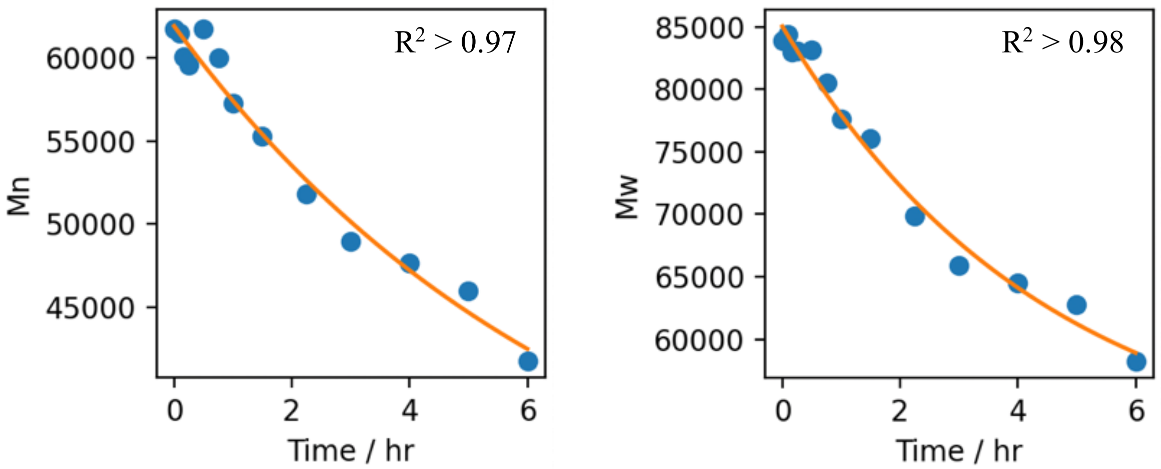

Figure S61. M_n and M_w values of **Table 1** entry **2a** over six hours of ultrasonic degradation fitted to exponential decay curves, corresponding to a degradation rate of 0.1339 min^{-1} (M_n) and 0.2008 min^{-1} (M_w).

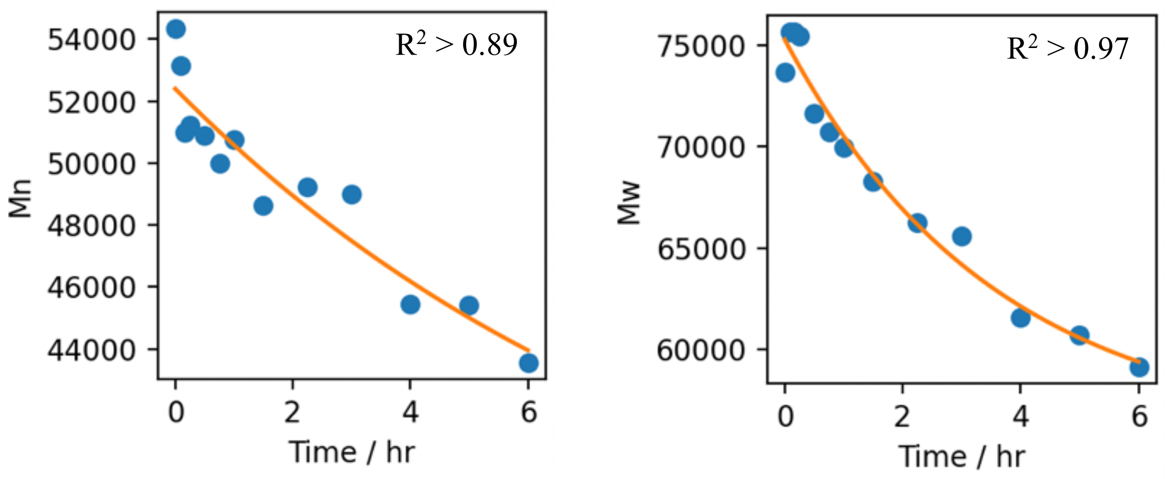

Figure S62. M_n and M_w values of **Table 1** entry **2b** over six hours of ultrasonic degradation fitted to exponential decay curves, corresponding to a degradation rate of 0.1040 min^{-1} (M_n) and 0.2440 min^{-1} (M_w).

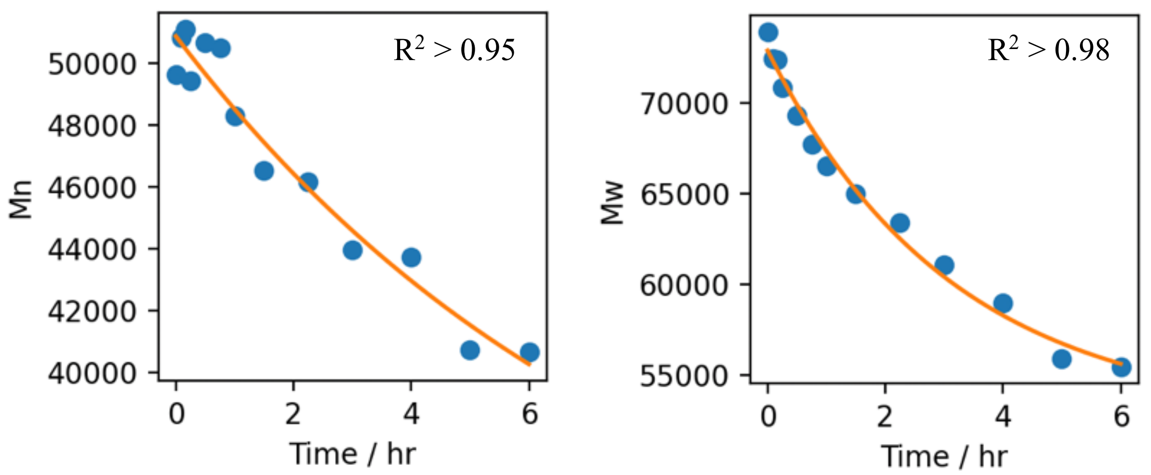

Figure S63. M_n and M_w values of **Table 1** entry **2c** over six hours of ultrasonic degradation fitted to exponential decay curves, corresponding to a degradation rate of 0.1176 min^{-1} (M_n) and 0.2729 min^{-1} (M_w).

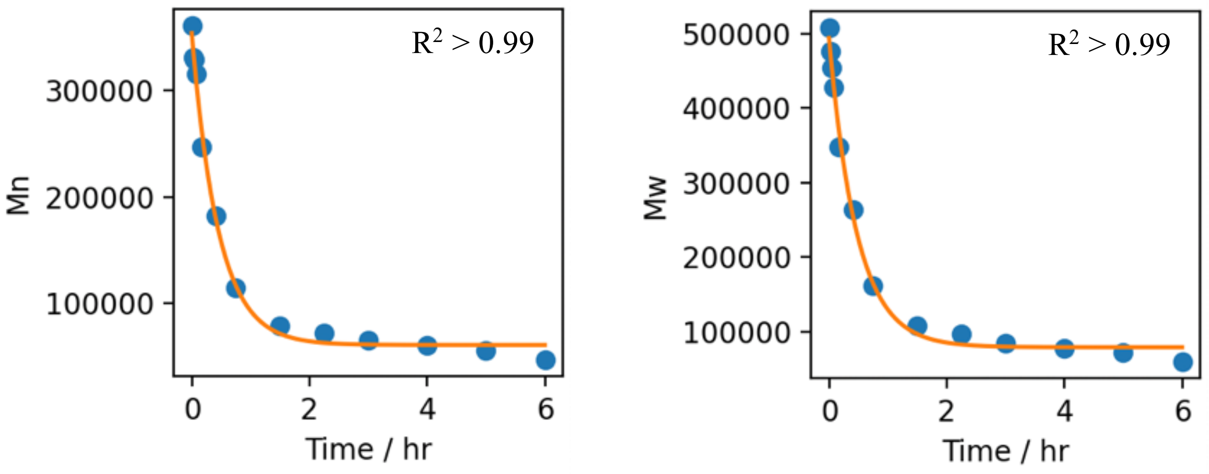

Figure S64. M_n and M_w values of **Table 1** entry **3a** over six hours of ultrasonic degradation fitted to exponential decay curves, corresponding to a degradation rate of 0.8904 min^{-1} (M_n) and 0.8782 min^{-1} (M_w).

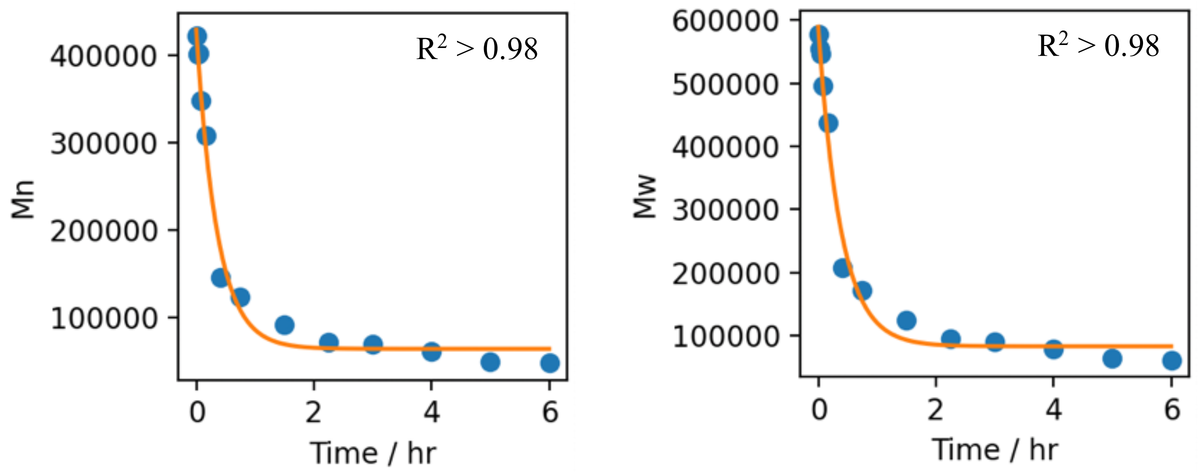

Figure S65. M_n and M_w values of **Table 1** entry **3b** over six hours of ultrasonic degradation fitted to exponential decay curves, corresponding to a degradation rate of 0.9401 min^{-1} (M_n) and 0.9262 min^{-1} (M_w).

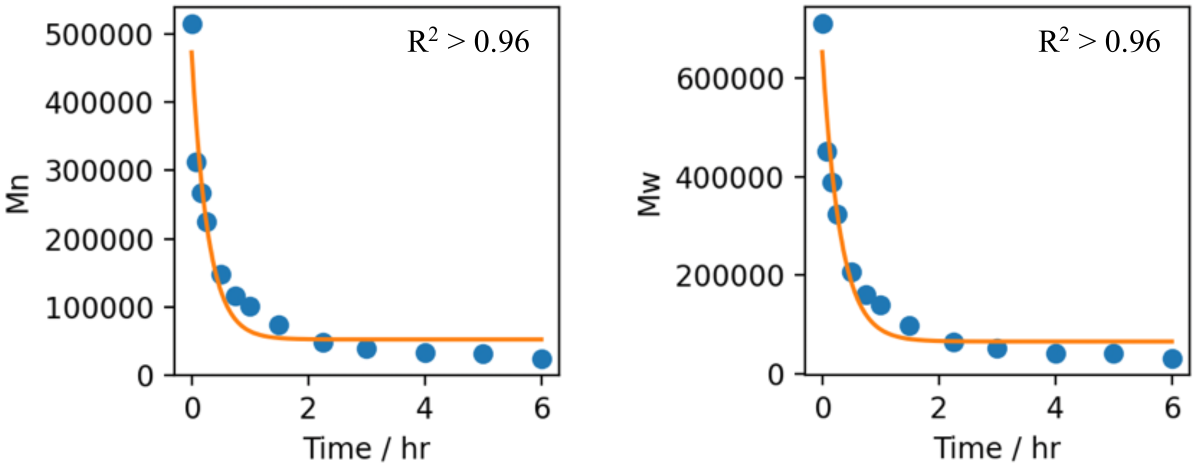

Figure S66. M_n and M_w values of **Table 1** entry **3c** over six hours of ultrasonic degradation fitted to exponential decay curves, corresponding to a degradation rate of 0.9712 min^{-1} (M_n) and 0.9579 min^{-1} (M_w).

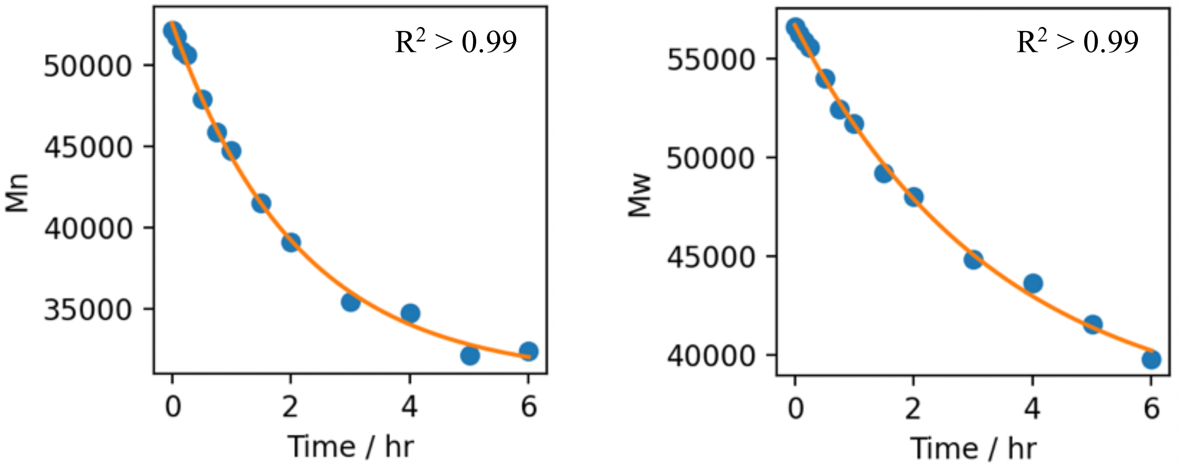

Figure S67. M_n and M_w values of **Table 1** entry **4a** over six hours of ultrasonic degradation fitted to exponential decay curves, corresponding to a degradation rate of 0.3770 min^{-1} (M_n) and 0.2524 min^{-1} (M_w).

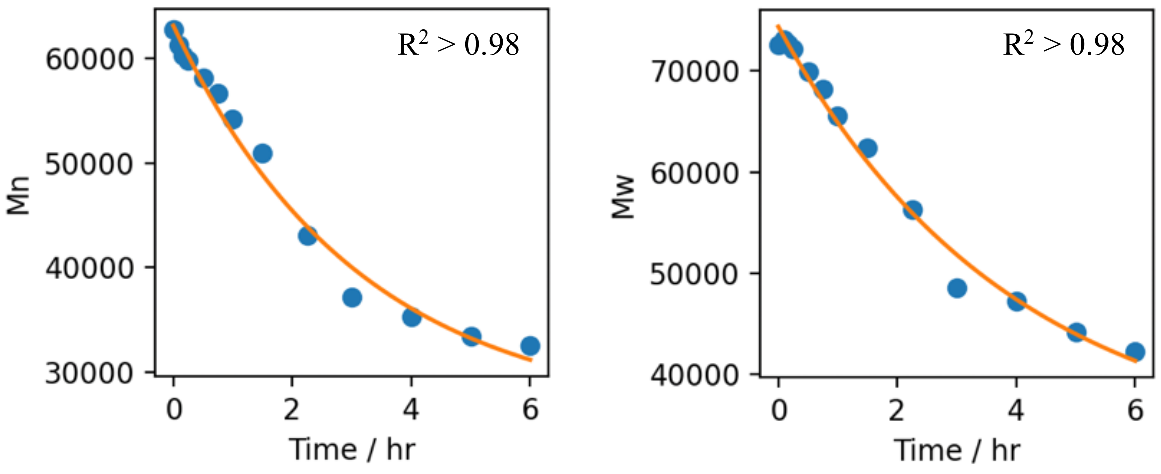

Figure S68. M_n and M_w values of **Table 1** entry **4b** over six hours of ultrasonic degradation fitted to exponential decay curves, corresponding to a degradation rate of 0.2740 min^{-1} (M_n) and 0.2252 min^{-1} (M_w).

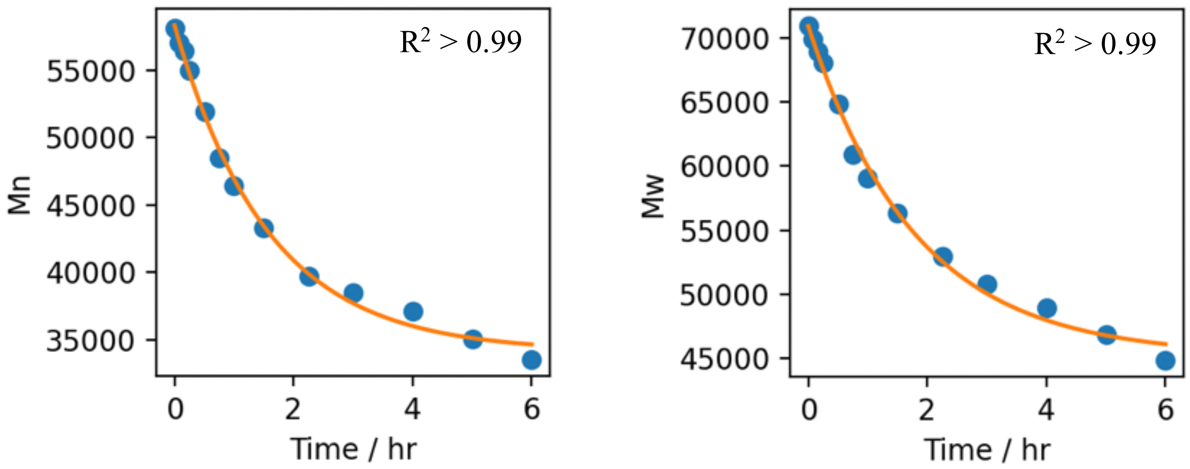

Figure S69. M_n and M_w values of **Table 1** entry **4c** over six hours of ultrasonic degradation fitted to exponential decay curves, corresponding to a degradation rate of 0.4711 min^{-1} (M_n) and 0.4262 min^{-1} (M_w).

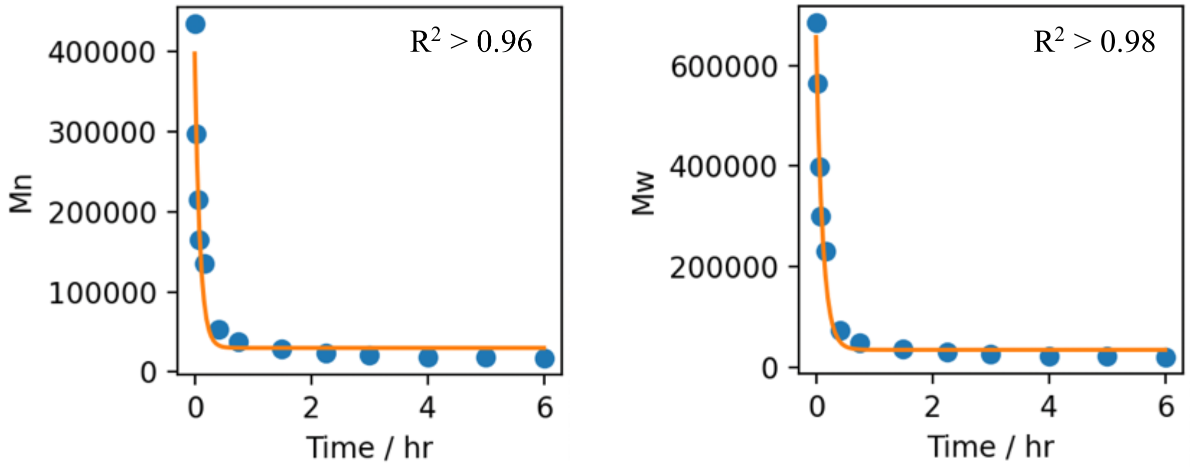

Figure S70. M_n and M_w values of **Table 1** entry **5a** over six hours of ultrasonic degradation fitted to exponential decay curves, corresponding to a degradation rate of 0.9999 min^{-1} (M_n) and 0.9998 min^{-1} (M_w).

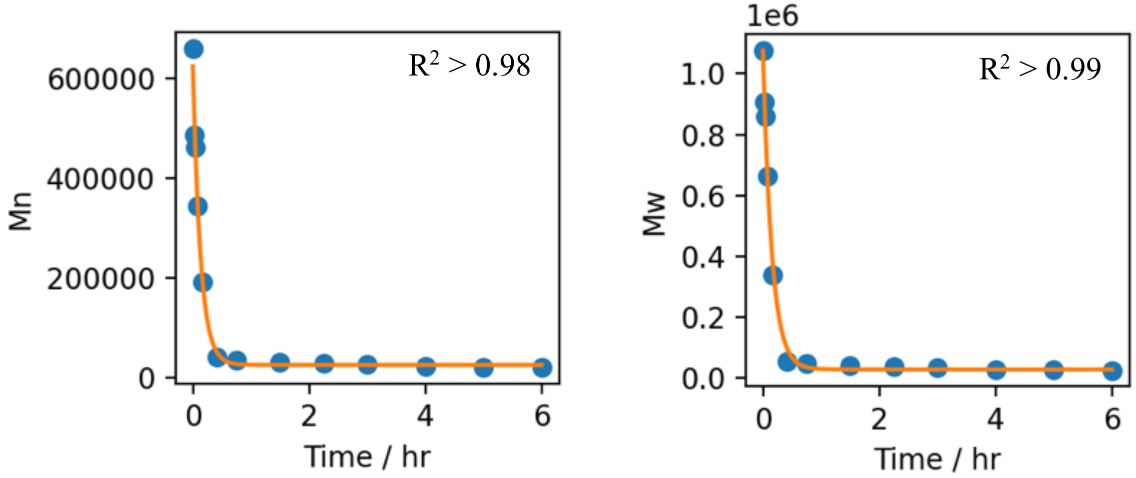

Figure S71. M_n and M_w values of **Table 1** entry **5b** over six hours of ultrasonic degradation fitted to exponential decay curves, corresponding to a degradation rate of 0.9995 min^{-1} (M_n) and 0.9986 min^{-1} (M_w).

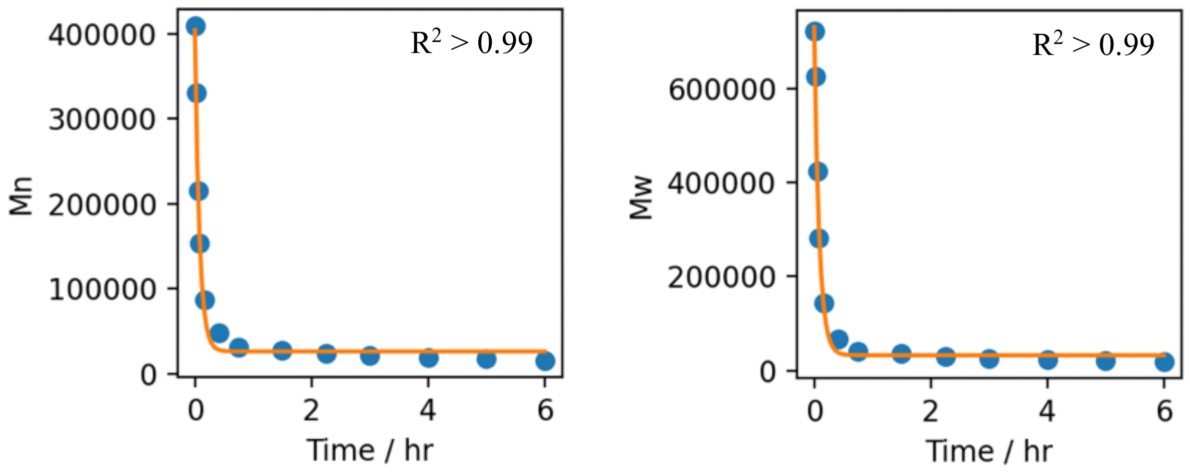

Figure S72. M_n and M_w values of **Table 1** entry **5c** over six hours of ultrasonic degradation fitted to exponential decay curves, corresponding to a degradation rate of 0.9999 min^{-1} (M_n) and 0.9999 min^{-1} (M_w).

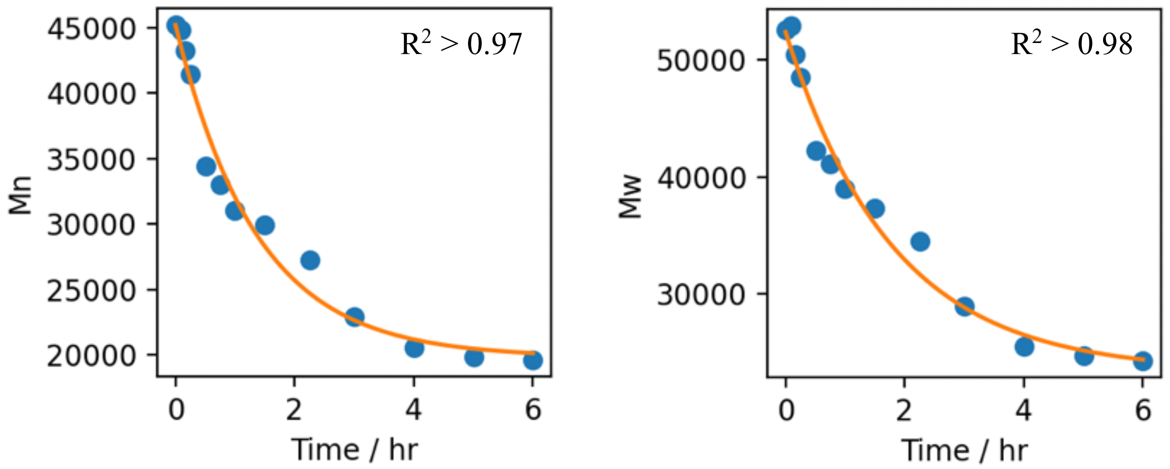

Figure S73. M_n and M_w values of **Table 1** entry **6a** over six hours of ultrasonic degradation fitted to exponential decay curves, corresponding to a degradation rate of 0.5188 min^{-1} (M_n) and 0.4258 min^{-1} (M_w).

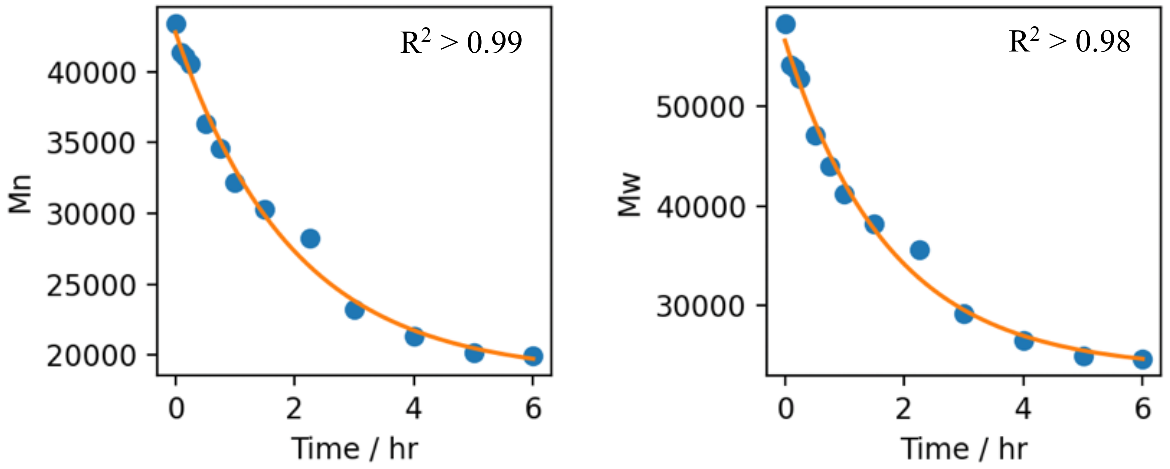

Figure S74. M_n and M_w values of **Table 1** entry **6b** over six hours of ultrasonic degradation fitted to exponential decay curves, corresponding to a degradation rate of 0.3998 min^{-1} (M_n) and 0.4337 min^{-1} (M_w).

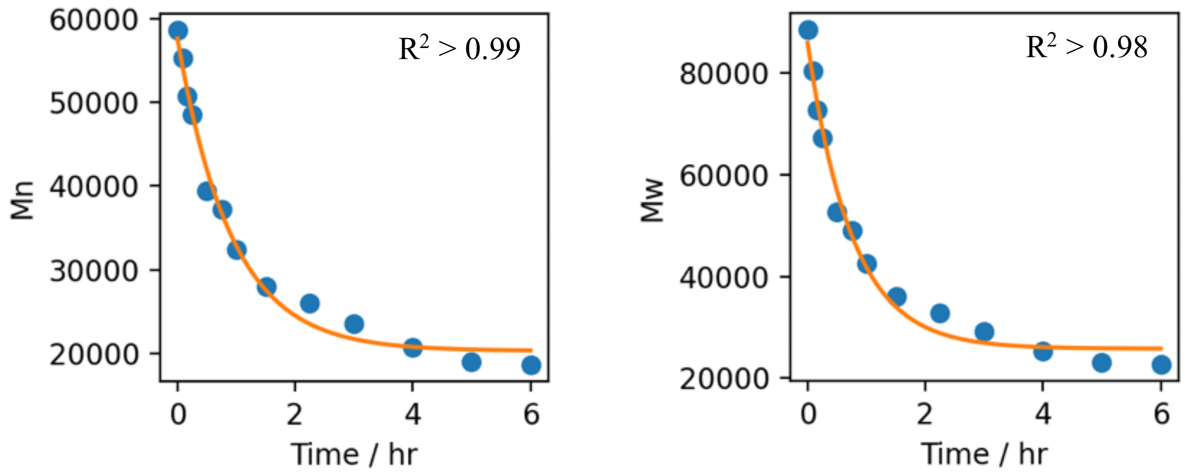

Figure S75. M_n and M_w values of **Table 1** entry **6c** over six hours of ultrasonic degradation fitted to exponential decay curves, corresponding to a degradation rate of 0.6638 min^{-1} (M_n) and 0.7340 min^{-1} (M_w).

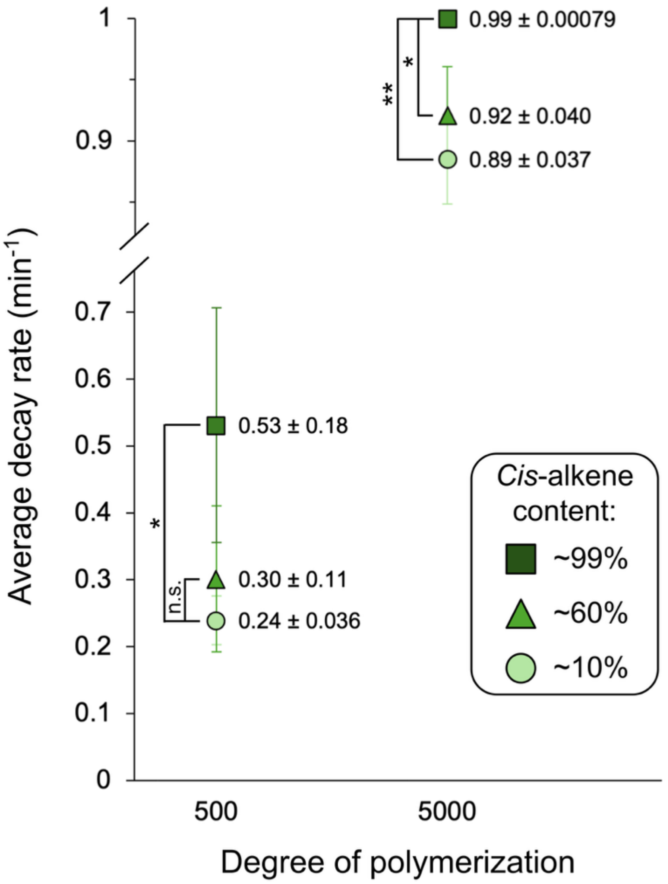

Figure S76. Average polynorbornene ultrasonic degradation rates as a function of degree of polymerization and *cis*-alkene content. Degradation rates were extracted from exponential decay fits to the M_w values over six hours of ultrasonic degradation. Data are presented as the average of triplicate measurements from three distinct samples (a-c of each numbered entry in Table 1) with error bars representing the standard deviation of the mean. Statistical analysis was performed with a Welch's t-test of paired samples: n.s. $p > 0.1$, * $p \leq 0.1$, ** $p < 0.05$.

6. References

(1) Love, J. A.; Morgan, J. P.; Trnka, T. M.; Grubbs, R. H. A Practical and Highly Active Ruthenium-Based Catalyst That Effects the Cross Metathesis of Acrylonitrile. *Angew. Chem. Int. Ed.* **2002**, *41* (21), 4035–4037. https://doi-org.ccl.idm.oclc.org/10.1002/1521-3773(20021104)41:21<4035::AID-ANIE4035>3.0.CO;2-I.

(2) Lenhardt, J. M.; Black Ramirez, A. L.; Lee, B.; Kouznetsova, T. B.; Craig, S. L. Mechanistic Insights into the Sonochemical Activation of Multimechanophore Cyclopropanated Polybutadiene Polymers. *Macromolecules* **2015**, *48* (18), 6396–6403. https://doi.org/10.1021/acs.macromol.5b01677.
